# Species-Specific Transcription Factors Associated with Long Terminal Repeat Promoters of Endogenous Retroviruses: A Comprehensive Review

**DOI:** 10.3390/biom14030280

**Published:** 2024-02-26

**Authors:** Md Jakir Hossain, Perpetual Nyame, Kazuaki Monde

**Affiliations:** Department of Microbiology, Faculty of Life Sciences, Kumamoto University, Kumamoto 860-8556, Japan; 217r6121@st.kumamoto-u.ac.jp (M.J.H.); 214r5156@st.kumamoto-u.ac.jp (P.N.)

**Keywords:** endogenous retrovirus, transcription factor, long-terminal repeat

## Abstract

Endogenous retroviruses (ERVs) became a part of the eukaryotic genome through endogenization millions of years ago. Moreover, they have lost their innate capability of virulence or replication. Nevertheless, in eukaryotic cells, they actively engage in various activities that may be advantageous or disadvantageous to the cells. The mechanisms by which transcription is triggered and implicated in cellular processes are complex. Owing to the diversity in the expression of transcription factors (TFs) in cells and the TF-binding motifs of viruses, the comprehensibility of ERV initiation and its impact on cellular functions are unclear. Currently, several factors are known to be related to their initiation. TFs that bind to the viral long-terminal repeat (LTR) are critical initiators. This review discusses the TFs shown to actively associate with ERV stimulation across species such as humans, mice, pigs, monkeys, zebrafish, Drosophila, and yeast. A comprehensive summary of the expression of previously reported TFs may aid in identifying similarities between animal species and endogenous viruses. Moreover, an in-depth understanding of ERV expression will assist in elucidating their physiological roles in eukaryotic cell development and in clarifying their relationship with endogenous retrovirus-associated diseases.

## 1. Introduction

**Figure 1:**
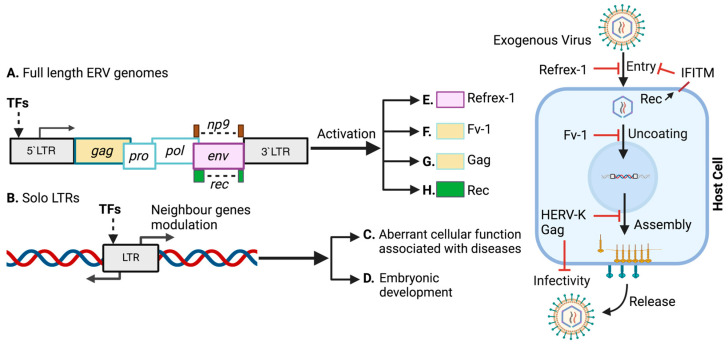
ERVs protect the host cells from the infection of exogenous retroviruses. (**A**) ERVs encode *gag*, *pro*, *pol*, *env*, and accessory genes *rec/np9*. (**B**) The solo LTRs lose the viral genes between 5′LTR and 3′LTR. (**C**) The solo LTRs modulate the genes associated with several diseases. (**D**) The solo LTRs modulate the genes associated with the placental and preimplantation developments. (**E**) The refrex-1 inhibits the viral entry in feline cells. (**F**) The Fv-1 inhibits the post-entry in murine cells. (**G**) The HERV-K Gag inhibits the viral assembly and viral infectivity. (**H**) The HERV-K Rev induces the IFITM expression to inhibit the viral infection. The graphic was created with BioRender.com. TFs: transcription factors; HERV-K: human endogenous retrovirus-K.

Retroviruses are representative examples of viruses that exist both endogenously and exogenously [1,2,3]. For example, human endogenous retrovirus-K (HERV-K) integrates its proviral DNA, which consists of four coding regions (*gag*, *pro*, *pol*, and *env*) and an accessory gene (*rec* or *np9*) flanked by long terminal repeats (LTRs), into the human genome [4,5] (Figure 1A). By incorporating their DNA sequences into the germline [5,6], they eventually become generationally inheritable endogenous retroviruses (ERVs) [7,8,9] and are subject to natural selection according to the Mendelian formula. As a result, their adverse effects are eliminated or inactivated by mutations over time [10]. In contrast, some ERVs, such as HERV-K, may still produce infectious virus-like particles [11,12,13,14,15,16]. Although ERVs remain as fossils millions of years ago, they constitute ~8.3% of the human genome [17,18,19,20,21,22].

Generally, ERVs are expressed in the placenta and embryonic tissues [23,24,25,26,27], certain cancer cells [28,29,30,31,32,33,34,35,36,37,38,39,40,41,42,43,44,45], as well as patients with schizophrenia [46,47,48,49], rheumatoid arthritis [50,51,52,53], and amyotrophic lateral sclerosis (ALS) [54,55]. Transcription factors (TFs) are the main factors involved in ERV expression in the placenta; however, multiple oncogenic signaling pathways, such as the WNT signaling pathway, are also important for ERV expression [56]. Thousands of ERVs are transcribed in somatic tissues at multiple sites. These ERV expressions in somatic tissues are leaky or driven by ERV LTRs [57]. The induction of HERV-K expression by viral infection may contribute to a variety of illnesses [58,59]. Notably, infection with Kaposi sarcoma-associated herpes virus triggers the expression of HERV-K envelope-derived proteins (Rec and NP9), resulting in viral propagation, which contributes to cellular dysfunction [60]. Furthermore, ERVs can recombine to generate solo-LTR that can induce the expression of neighboring genes [61,62] (Figure 1B). Once activated, ERVs are expressed in healthy tissues and act as potential oncogenic drivers [63,64,65]. Consequently, they function as ancillaries in processes that aid the progression of diseases, such as oncogenesis, and cellular senescence. Moreover, it may trigger diseases such as autoimmune diseases and neurological disorders [66,67,68,69,70] (Figure 1C).

ERV LTRs may serve as transcription initiation sites for their open reading frames (ORFs) through multiple cellular mechanisms [71,72,73]. Therefore, the *cis*-regulatory elements of ERV LTRs are utilized in physiological processes, including stem cell pluripotency, proliferation, and survival [27,73,74,75,76,77,78,79,80,81] (Figure 1D). Moreover, ERVs serve as host defense against exogenous retroviruses [23,82,83,84,85,86,87,88,89,90,91,92,93] (Figure 1E–H). Presumably, a wide range of TF-binding motifs routinely regulate LTR activation, which is required for triggering HERVs, murine endogenous retroviruses (MERVs), porcine endogenous retroviruses (PERVs), simian endogenous retroviruses (SERVs), zebrafish endogenous retroviruses (ZFERVs), and yeast retrotransposons (Ty1/3) [94,95].

**Figure 2:**
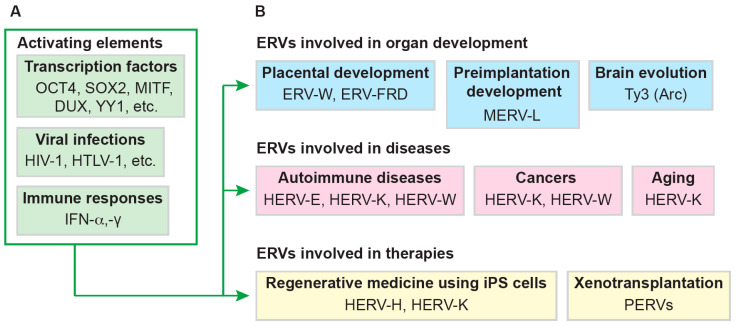
ERV-associated diseases, ERV evolution, and therapeutics. (**A**) ERVs transcription is activated by several factors such as transcription factors, viral infections, and immune responses. (**B**) The activation of ERVs is crucial for the placental development and preimplantation development. The ERVs expression is upregulated in several diseases. In addition, controlling the ERVs expression must be required for the regenerative medicine and xenotransplantation. MITF: microphthalmia-associated transcription factor; YY1: Yin Yang 1; HIV-1: human immunodeficiency virus type 1; HTLV-1: human T-lymphotropic virus type 1; IFN: interferon; ERV: endogenous retrovirus; HERV: human endogenous retrovirus; iPSCs: induced pluripotent stem cells; PERV: porcine endogenous retrovirus.

As demonstrated in earlier studies, TFs, or sequence-specific DNA-binding factors, are molecules that bind to a specific DNA sequence and modulate the transcription of DNA into mRNA [96,97]. In multicellular organisms, TFs are pivotal for cellular differentiation, and disease progression [98,99]. TFs possess species-specific DNA-binding domains, which facilitate their binding to target sequences [100]. Therefore, research on the uniqueness of TFs to LTRs, the variety of LTRs in several species, and the positioning of LTRs in the genome are indispensable for unraveling the mystery of ERVs. Currently, ERV LTR-activated TFs and LTR-driven genes have been numerously reported based on the reporter assay in vitro, the next-generation sequencing analysis, and the bioinformatic analysis as described below (Figure 2A). However, a great deal of information that has been exhaustively analyzed must be organized for new discoveries in future. This review highlights impactful data depicting different ERV LTRs activation by TFs as well as associated cellular dysfunctions across different species. We hope that this review will serve as a comprehensive resource for the practical application of regenerative medicine using induced pluripotent stem cells (iPSCs) and xenotransplantation using porcine tissue, as well as for the development of cancer and aging research (Figure 2B). We also hope that the coexistence of the host with the virus will provide clues to elucidate how the virus has contributed to biological evolution.

## 2. HERV LTRs Are Required for the Transcription of Neighboring Genes

LTRs are essential for transcription in multicellular organisms [71,72,73]. Based on the LTR sequence, HERV-K (HML-2) LTR is classified into LTR5A, LTR5B, and LTR5Hs subtypes [101]. Indeed, the HERV-K core promoter elements (U3, R, and U5) are essential to make the promoter functional [102,103,104]. As every provirus has undergone distinct mutations over the course of millions of years, these LTR sequence modifications may affect the binding of TFs to LTR-binding sites, which in turn may lead to the distinctive expression of HML-2 [105]. Chromatin state analysis has revealed that different HERV LTRs enriched in the enhancer regions are distinct across cell types [106]. The expression of neighboring genes from ERV LTR was analyzed by the activation/silencing of the HERV-K LTR5Hs using CARGO with CRISPR activation (CRISPRa) or interference (CRISPRi) [62]. The results indicated that the chromatin state of its neighboring genes is remarkably changed and that LTR5Hs acts as distal enhancer or suppressor to regulate the expression of at least 275 genes [62]. Furthermore, the expression of neighboring genes, which are located either proximal or distal within a range of ~200 kb from LTR5Hs, is regulated by LTR5Hs activation or silencing [62]. In summary, because LTRs affect the expression of neighboring genes within a 200 kb range, identifying the elements involved in transcription from the LTRs may allow us to determine causal relationships with various ERV-related diseases.

### 2.1. TFs Associated with LTR Activation of HERV-K

p53 directly upregulated the promoter activity of LTR5Hs rather than LTR5A and LTR5B in cervical carcinoma (HeLa) and HEK293T cells [101]. Luciferase reporter and ChIP assay results proved that the two binding sites for p53 on LTR5Hs are important for upregulating transcription and activating other LTRs [101]. Although p53 plays an important role in suppressing cancer progression, the mechanism by which it activates HERV-K remains unclear. A recent study reported that the presence of anti-HERV-K Env antibodies is important for lung cancer immunotherapy [45]. Thus, if p53-mediated HERV-K activation is involved in lung cancer immunotherapy, this will be a topic for future research.

HERV-K LTR5Hs (HERV-K113) has a full-length ORF and is located on chromosome 19p13.11 [14]. The prevalence of HERV-K113 mRNA is higher (29%) than that of other HERVs in various cancer cells, particularly teratocarcinoma cells [14]. Specific TFs are necessary to activate this LTR-driven genome, which include Sox2, Oct4, and Nanog (Table 1) [23,107,108]. However, the individual expression of Oct4 and Nanog is insufficient to activate HERV-K LTR5Hs [107], while the combined expression of Oct4 and Nanog, along with Sox2, markedly enhances the transactivation capability [107]. Historically, Sox2 has been shown to control cancer stem cell maintenance and self-renewal, fostering oncogenic signaling [109,110,111]. Interestingly, HERV-K expression is significantly upregulated in germ cell tumors, melanomas, and ovarian cancers compared to that in healthy tissues [42,112,113,114]. These findings suggest that the transactivation of HERV-K LTR5Hs by Sox2 is involved in numerous malignant tumors.

Double homeobox 4 (DUX4) is another prominent TF that activates HERV-L LTRs in rhabdomyosarcoma cells (muscle cancer cells) [115,140,141,142]. Indeed, ERV transcripts were detected in the skeletal muscles of individuals diagnosed with facioscapulohumeral muscular dystrophy (FSHD) [115,142]. RNA-seq and ChIP assay results suggest that DUX4 overexpression causes the initial manifestation of FSHD [115]. Notably, DUX4 induces HERV-K LTR5Hs on chromosome 7p22 in glioblastoma and myoblastoma cell lines (Table 1), which may result in neurological diseases [17,115].

The resurrection of HERV-K LTRs is alarming for genomic stability and may be associated with the initiation and upregulation of renal cell carcinoma (RCC) [116,143,144,145]. Numerous studies have reported the expression of different HERVs in patients with RCC [146]. In patients with RCC, HERV-K LTR5Hs LTR is located on the long arm of chromosome 6, and HERV-E LTR is significantly expressed in the presence of hypoxia-inducible transcription factor (HIF), which binds to transcriptionally active LTR elements [146]. Thus, evidence suggests that HIF-dependent reactivation of dormant promoters embedded within endogenous retroviral LTRs is a potential contributing factor to dysregulated gene expression in RCC [116].

Previous research on the binding motifs of TFs revealed the presence of microphthalmia-associated transcription factor-M (MITF-M) on HERV-K LTR as well as some other retroviral LTRs [123]. The expression of HERV-K on chromosome 1 was shown to be significantly higher in the presence of MITF-M in melanoma and HEK293 cells [123,147]. Further analysis uncovered that the sequence of MITF-M (MITF-1, MITF-2, and MITF-3), TATA, and Inr is quite conserved (5′CACATG3′) in over a hundred HERV-K LTRs [123]. Mutants of the MITF-1, -2, or -3 motifs at the HERV-K LTR were unable to initiate transcripts in malignant melanoma cell lines (MeWo cells), which express large amounts of endogenous MITF-A and -M [123]. MITF-M binds to the LTR at the 5′ and 3′ ends, activating both HERV-K LTRs [123,147,148]. This suggests that MITF-M alters the expression of neighboring genes in malignant melanoma cells.

HERV-K env RNA expression is upregulated in patients with atypical teratoid rhabdoid tumor (AT/RT) by deletion or mutation of integrase interactor 1 (SMARCB1) [95]. Based on computational assessment using the PROMO software v8.3 of TRANSFAC, c-Myc protein binding sites were identified within the HML-2 LTR5Hs sequences [149]. SMARCB1 is a transcriptional repressor of HIV-1 LTR [150]. The active LTRs in loci 7p22.1a and 7p22.1b are highly expressed in patients with AT/RT as a result of c-Myc binding to the HERV-K LTR (Table 1) [95]. Similar results were found in 293T cells [150], suggesting that SMARCB1 is a repressor and c-Myc is an activator for HERV-K LTR in AT/RT.

HERV-K is actively involved in breast cancer progression in the presence of female sex hormones estradiol and progesterone [118]. Although estradiol and progesterone have other biological functions, they synergistically activate HERV-K through their receptors in T47D human breast cancer cells [118]. Electrophoretic mobility shift assay (EMSA) and co-immunoprecipitation assay showed that the progesterone receptor (isoform B) binds to the progesterone response element within LTR5Hs [118]. Conversely, overexpression of Oct4 significantly (two-fold) enhanced HERV-K10 transcription, and progesterone treatment synergistically activated HERV-K10 LTR in primary mammary epithelial cells [118]. Thus, it can be assumed that HERV-K, which is activated by female sex hormones, drives breast cancer progression [118].

The TF Yin Yang 1 (YY1) is highly conserved from African clawed frogs (*Xenopus laevis*) to humans [151,152]. It is ubiquitously expressed in pluripotent differentiated cells, mouse teratocarcinoma cells (F9), mouse fibroblasts (NIH3T3), rat embryo fibroblasts, and HeLa cells [153,154]. YY1, together with different cofactors, mediates the activation, repression, or initiation of transcription in ERVs [155]. YY1 binds to a motif within 62nt-83nt of the HERV-K LTR5Hs and acts as an enhancer-binding protein in human teratocarcinoma (GH and Tera2), hepatocarcinoma (HepG2), and HeLa cells [120]. Additionally, YY1 enhancer complexes activate HERV-K LTR5Hs by binding at the 5′ end of the U3 region [120]. In contrast, YY1 induces silencing of exogenous and endogenous retroviruses by recruiting tripartite motif-containing protein 28 (TRIM28) and its complex in mouse embryonic cells [121,156,157,158,159]. Thus, ERV LTR is activated or suppressed depending on the transcriptional activator or repressor that binds to YY1; therefore, it is important to investigate the relationship between YY1-binding cofactors and diseases.

HERV-K is also associated with numerous neural diseases [55]. HERV-K env leads to neuronal injury in the presence of the transactive response DNA-binding protein 43 (TDP-43) [119]. The co-expression of HERV-K RT and TDP-43 proteins has been observed in most neurons, with a significant positive correlation between them [160]. Five different TDP-43 binding sites were found on the HERV-K LTR, which regulate its activation [119]. Human neural cells transfected with TDP-43 showed enhanced HERV-K expression, whereas knockdown of endogenous TDP-43 resulted in decreased HERV-K expression [119]. In addition, the levels of antibody against HERV-K were markedly increased in patients with ALS, multiple sclerosis, and Alzheimer’s disease [161]. Moreover, specific post-translational modifications of TDP-43 may affect HERV-K expression patterns. For example, formation of TDP-43 aggregates alters HERV-K RT expression and cellular localization of viral proteins [117]. Misfolded TDP-43 is aggregated and transmitted in patients with ALS [162]. Interestingly, prion-like proteins (e.g., yeast Sup35 prion NM domain and Tau microtubule-binding domain) are transmitted by ERVs such as HERV-K and HERV-W [70]. Thus, if ERVs form particles and become active in other cells by transmitting TFs listed in Table 1, including TDP-43, they may be involved in diseases such as ALS.

HERV-K LTRs and HERV-E.PTN are TATA-independent promoters regulated by three GC boxes that serve as binding sites for Sp1 and Sp3 [103,163]. Sp1/Sp3 are TFs for TATA-less promoters that are available early after zygote formation [164]. HERV-K LTR activation was reduced by approximately 20% and 50% following knockdown of Sp1 and Sp3, respectively, in human melanoma (Mel-C9) and teratocarcinoma (GH) cell lines [103]. Mutation of the Sp1-binding motif (451–462 nt) in HERV-E.PTN markedly reduced transcriptional activity in choriocarcinoma cells of the fetal placenta (JEG-3, BeWo, and JAR). Since HERV-W and HERV-FRD Env play critical roles in placental development [130], Sp1 and Sp3 may trigger HERV-W transcription in placental cells. However, the active HERV-W LTR has a mutation in the Sp1-binding motif, while the inactive HERV-W LTR has an intact Sp1-binding motif in lung fibroblasts (LC5) (Table 1) [129]. Thus, transcriptional activation by Sp1 and Sp3 is complex, and their regulatory mechanisms may differ depending on other transcription elements and cell lines.

### 2.2. Viral Infection Activates HERV-K and HERV-W Expression

The HTLV-1 Tax protein, detected in patients with myelopathy or tropical spastic paraparesis (HAM/TSP), is a powerful trans-activator capable of inducing many cellular genes through its activation domains [165]. HTLV-1 Tax trans-activator could activate the LTRs of different HERV families (HERV-W8, HERV-H, HERV-K, and HERV-E) in T cell lines [125]. Indeed, various Tax mutants have been shown to affect LTR activation to different degrees. Moreover, modulation of these LTRs by Tax activated CREB and possibly NF-κB in Jurkat cells, which in turn may positively regulate the transcription of HERV genes or other proximal cellular genes in HTLV-1-infected patients [125].

Several TFs listed in Table 1, such as AP-1, CREB, CEBP (C/EBP_α_), c-Rel, NF-AT, CEBP_β,_ NF-κB (p50:p52), Rel-A, p53, YY1, c-Myc, Sp1, Sp3, and signal transducer and activator of transcription 1 (STAT1), potentially interact with HERV-K LTRs. Interestingly, multiple NF-κB sites are present in the HERV-K promoter [166,167]. Luciferase reporter gene and ChIP assay analyses showed that NF-κB mediates the activation of HERV-K via HIV-1 Tat protein [122]. As the HERV-K LTR contains potential NF-AT binding sites, NF-AT activation could additionally contribute to HERV-K Tat-driven expression and might compensate for the absence of NF-κB activity. These reports suggest that both NF-κB and NF-AT activation in response to HIV-1 Tat drives transcription from the HERV-K promoter in HIV-1-infected patients [122]. HIV-1 release and infectivity are reduced by coassembly between HIV-1 Gag and HERV-K Gag in the HERV-K Gag-overexpressing cells [83,84] (Figure 1G). The endogenous retroviruses might be activated by the infection of exogenous retroviruses to protect the host cells from exogenous retroviral threats.

LTR-driven HERV-W transcription is activated by herpes simplex virus type 1 (HSV-1) immediate early protein (IE1) through the cellular TF Oct-1 [128]. In addition, anti-HERV-W antibody level is elevated with anti-Epstein–Barr virus antibody in the patients with autoimmune demyelinating disorders [168]. Two Oct-1-binding motifs are conserved in the HERV-W LTR series [129]. Specifically, few HERV-W LTRs are stimulated in the presence of IE1, which potentially upregulates the expression of other genes [128]. Furthermore, HSV-1 immediate early protein (ICP0) increases HERV-K transcription via the AP-1-binding motif in the LTR [169]. Therefore, the relationship between various HSV-1-associated diseases and HERVs is a topic for future research.

Subsequent research has clarified that HERV-W LTR is also activated by influenza A/WSN/33 infection [170]. Although its underlying mechanism remains to be elucidated, the induction of HERV-W was found to depend on the cell line, because the expression of HERV-W *gag* and *env* genes was relatively enhanced in influenza-infected CCF-STTG1 and U937 cells, but not in 293F cells. Notably, interferon beta (IFN-β) level positively correlated with HERV-W expression in the infected cells [170]. This suggests that IFN-induced HERVs expression cannot be disregarded.

### 2.3. Interferon-α, and γ Trigger HERV-K Expression

Superantigen (SAg) IDDMK_1,2_ 22, associated with type-1 diabetes, is derived from HERV-K18 mapped on chromosome 1q21.2-q22 [171]. IFN-α treatment upregulated the expression of SAg and HERV-K18 in T cells, while a cocktail treatment of IFN-α and IFN-γ markedly increased HERV-K18 expression [126], suggesting that HERV-K18 is induced by IFN-α and this induction can be amplified by IFN-γ priming.

IFN-γ signaling-induced HERV-K102 expression has been demonstrated in various cell lines [124]. Expression of HERV-K genes was higher in patients with cutaneous leishmaniasis, and IFN-γ level is known be elevated in these due to leishmania parasite infection [124,172]. Transposase-accessible chromatin sequencing (ATAC-seq) and ChIP sequencing analyses showed the HERV-K102 expression was upregulated via solo-LTR LTR12F upon treatment with IFN-γ in HeLa cells [124]. IFN regulatory factor 1 (IRF1) and, potentially, STAT1 are conjugally recruited to activate LTR12F located upstream of HERV-K102 [124]. Upon activation of LTR12F along with the enhancer histone H3 dimethylation of lysine 4 (H3K4me2), HERV-K102 activates following IFN-γ signaling [124]. This suggests that HERV-K102 activation is sensitive to IFN-γ and is regulated through IRF1 recruitment followed by LTR12F activation. The notable finding is that HERV-K102 expression is upregulated by utilizing upstream solo-LTR rather than its own LTRs, and that IFN-γ and IRF1 recruitment are the trigger for solo-LTR activation.

## 3. TFs Associated with LTR Activation of Other HERVs

HERVs are classified according to the type of tRNA that binds to the primer binding site (PBS) located downstream of the 5′LTR. For example, in the case of HERV-K, HERV-K utilizes a lysine (K) tRNA, and in the case of HERV-W, it is tryptophan (W) tRNA [173].

### 3.1. HERV-E Is Activated by HIFs, Nuclear Factor of Activated T-Cells 1 (NFAT1), and Estrogen Receptor Alpha (ER-α)

HERV-E (CT-RCC-8 and CT-RCC-9), located on the long arm of chromosome 6 (GenBank accession number AL133408), is selectively expressed in most RCCs [174]. Notably, HERV-E Env protein has antigenic properties that immunologically provoke cytotoxic T cells to kill RCC cells both in vitro and in vivo [144,174]. HERV-E provirus was shown to be resurrected in the clear cell subtype of RCC (ccRCC) upon inactivation of the von Hippel–Lindau (*VHL*) gene, which is a tumor suppressor gene [131]. Furthermore, this activation of HERV-E can be stabilized by HIF-2α but not HIF-1α [131]. Computational analysis showed that the binding motif of HIF-2α is located on the HERV-E LTR, and in vitro investigation found a direct correlation between the expression levels of HIF-2α and HERV-E in ccRCC [131]. Furthermore, ChIP analysis revealed a direct binding association between HIF-2α and HERV-E 5′LTR [131]. Taken together, these findings suggest that of *VHL* suppression-activated HERV-E, which could be promoted and stabilized by HIF-2α in RCC [131].

Elevated ERV protein levels have also been found in patients with systemic lupus erythematosus (SLE) [175], and autoreactive CD4^+^ T cells play a principal role in this disease [176]. The TFs NFAT1 and ER-*α* bind to the HERV-E clone 4-1 LTRs located on chromosome 19p12 [132]. Overexpression of NFAT1, and ER-*α* activated the HERV-E clone 4-1 5′LTRs in CD4^+^ T cells of patients with SLE, as revealed by luciferase reporter and the ChIP assay analyses [132]. In contrast, the antisense RNA miR-302d transcribed from the 3′LTR of HERV-E clone 4-1 induces DNA hypomethylation and is associated with SLE [132]. The hypomethylation activity of HERV-E has been confirmed in patients with SLE by COBRA validation [177,178]. Moreover, studies have reported that HERV-E mRNA, but not HERV-K and HERV-W, is increased in CD4^+^T cells from patients with SLE, suggesting a crucial role for HERV-E in the development of SLE [179].

### 3.2. HERV-L Is Activated by the Hepatocyte Nuclear Factor (HNF-1)

Solo-LTR (MLT2Bs), a member of the HERV-L family, is a promoter of the human beta-1,3-galactosyltransferase 5 (*β3Gal-T5*) gene, which is involved in type 1 Lewis antigen synthesis [79]. The ERV-L LTR promoter is most active in the gastrointestinal tract and mammary glands [79]. HNF-1 binds to ERV-L LTR and acts as a TF [79]. Two predicted sites for HNF-1 binding were identified at nucleotide positions 7–21 and 33–46 using the TRANSFAC TF database [79]. HNF-1 is expressed in tissues where the LTR promoter is active, including the intestine, stomach, kidneys, liver, and thymus [180]. LTR-driven β3Gal-T5 is expressed in the mammary glands, small intestine, trachea, colon, thymus, stomach, kidneys, liver, and lungs [79]. Of the two HNF-1-binding sites in the HERV-L LTR, the second site (position 33–46) was more important for the specific activation of the LTR promoter in a colorectal cancer cell line (LoVo). HNF-1, therefore, represents a candidate TF responsible for the tissue-specific activation of the HERV-L LTR promoter in various cancer cells, such as colorectal cancer cells.

### 3.3. HERV-W Is Activated by Glial Cells Missing-a

Human glial cells missing-a/1 (GCM-a/1) and murine glial cells missing-a (mGCM-a) are placenta-specific TFs required for placental development [181,182]. The HERV-W env syncytin-1, positioned on chr7q21-chr7q22, is regulated by GCM-a in BeWo and JEG3 cells [183]. Binding motif analysis identified two GCM-a-binding sites (25538-25545, 28026-28033) upstream of the HERV-W 5′LTR (GenBank accession no. AC000064; 7q21-7q22). Therefore, GCM-a can easily transactivate syncytin-1 gene, especially in trophoblasts [130]. The close proximity between the GCM-a-binding site and the LTR ensures the formation of an integral syncytiotrophoblast layer in the placenta [130].

Notably, GCM1 expression is activated via the WNT signaling pathway [184], suggesting that GCM1 and WNT signaling upregulate HERV-W expression in placental cells. In contrast, the HERV-W env protein increased the proliferation and viability of immortalized human uroepithelial cells. Results of colony-formation experiments and in vivo tumor xenografts suggest that syncytin-1 overexpression, due to two mutations in the 3′-LTR (T142C and A277G), has oncogenic potential in the urothelial cell carcinoma (UCC) [127]. The T142C mutation favors the binding of TF c-Myb to HERV-W 3′-LTRs and upregulates syncytin-1 overexpression [127]. This suggests that further research on HERV-W is important not only to elucidate the biological evolution due to placentation, but also to explore its relationship with development of cancers, such as UCC.

### 3.4. HERV-H Is Activated by Sox2, Nanog, and Oct4

HERV-H is present in the human genome as 100 full-length copies and >1000 solo-LTR copies. The solo-LTR of HERV-H was integrated into a gasdermin-like protein (GSDML) located on chromosome 17q21 during hominoid evolution. This solo-LTR drives GSDML–GSDM gene transcription in human gastric and breast cancers [133,134]. The expression and promoter activity of HERV-H LTR depend on the cell type [129]. Moreover, the HERV-H LTR has different TF-binding sites, such as Sp1, GC box, and TATA box [135,136]. RNA-seq revealed a binding association between HERV-H and the TFs Sox2, Nanog, and Oct4. During differentiation of embryonic stem cells (ESC), this binding association was greater for Sox2 than that for Nanog or Oct4 [185]. Ectopic expression of LBP9, Oct4, Nanog, and Klf4 activated HERV-H transcription in human primary fibroblasts, whereas overexpression of Myc or Sox2 failed to activate HERV-H [137]. Disruption of HERV-H transcripts compromises self-renewal, suggesting an important role for HERV-H expression in pluripotency [137]. Interestingly, the HERV-H transcripts (ESRG) play a crucial role in the maintenance of human pluripotency in iPSCs [186]. In summary, further studies on the transcriptional regulation of HERV-H and the function of its transcripts are important for the development of regenerative medicine using iPSCs.

### 3.5. HERV-S71 (HERV-T) Is Activated by GATA4 and FOXA2

GATA4 and Forkhead box protein A2 (FOXA2) are two distinct TFs that drive the regulatory network of the endoderm [187,188]. Both TFs were upregulated during definitive endoderm (DE) differentiation of hESCs. LTR6B, a DE-specific enhancer of ERVs, contains GATA4- and FOXA2-binding motifs and flanks HERV-S71-int [138], which is classified as HERV-T [189]. GATA4 and FOXA2, which bind to LTR6B, activated neighboring genes located in the vicinity of ~50 kb in DE cells, as determined by ChIP seq. This was supported by the finding that FOXA2 depletion downregulated the expression of LTR6B in DE cells, as shown by ATAC-seq and H2K27ac ChIP-seq analyses [138]. Thus, GATA4 and FOXA2 play a critical role in activating neighboring genes by binding to ERV LTR6B [138].

### 3.6. Solo-LTR (ERV-9) Is Activated by GATA-2, NF-Y, and MZF1

The ERV-9 solo-LTR is present upstream of the DNase I-hypersensitive site 5 (HS5) in the human β-globin locus control region and acts as an elite enhancer of cis-linked genes in the oocytes and progenitor stem cells [190]. EMSA revealed the binding motifs of GATA-2, ubiquitous NF-Y, and hematopoietic MZF1 within the ERV-9 solo-LTR in the erythroid cell line K562 [139]. NF-Y binds to the CCAAT motif on the ERV-9 LTR and recruits MZF1 and GATA-2 to form a complex [139]. This complex stabilizes their binding to neighboring GTGGGGA and GATA motifs. Subsequently, NF-Y binds to the complex, assembling an efficient LTR enhancer complex, which can accelerate the transcription of the β-globin gene [139].

## 4. TFs Associated with ERV LTR Activation in Other Species

As described HERV-K above, MERV-L LTR is also required for the transcription of two-cell embryo genes in mice [191]. Interestingly, the knockdown of MERV-L expression reduces the inner cell mass (ICM) genes (Oct4, Sox2, and Nanog) and trophectoderm differentiation genes (Tead4, Tcfap2c, and Cdx2) [192]. These findings suggest that ERVs, what are expressed during embryo, might be key factors for preimplantation development. On the other hand, the interference between ERV and exogenous retroviruses has also been highlighted in mice and cats. For instance, Friend-virus-susceptibility-1 (*Fv-1*), which is located on mouse chromosome 4 [193], restricts the murine leukemia virus (MLV) replication in mouse embryo cells [90,91] and determines the tropisms (MLV-N or MLV-B) [194] (Figure 1F). The *Fv-1* sequence has 60% identity with HERV-L *gag* gene [195]. The host defense of ERVs might have persisted through the process of biological evolution.

### 4.1. TFs Associated with LTR Activation of MERVs

Histone and DNA methylation result in the transcriptional suppression of MERVs during the initial phase of embryogenesis [196,197,198,199,200,201,202,203,204]. TRIM28, also called KRAB-associated protein 1 (KAP1), reportedly restricts gene expression via synergy between NuRD histone deacetylase complex, heterochromatin protein 1 (HP1), and histone methyltransferase SETDB1 [205]; however, to date, only a handful of its regions have been identified [206]. Nevertheless, it has been determined that the inhibitory influence of KAP1 on ERV expression can be manifested in three different ways. One way is that KRAB-zinc finger protein (ZFP809) binds to PBS of MERV and recruits the TRIM28 [207,208]. The TRIM28 recruitment is key for the silencing of endogenous retroviruses in mouse ESCs [208,209]. The other way is that KRAB-zinc finger protein (ZFP708) also binds to TRIM28 and plays a key role for the embryonic development via the ERV-K elements (RMER19B) silencing [210]. Last, YY1 binds to LTR and recruits the TRIM28 as described above [121,156,157,158,159]. On the other hand, TRIM28 can not only inactivate a gene during the initial events of embryogenesis, but also erase the murine leukemia virus in embryonic cells [207,208,211,212]. The loss of KAP1 causes remarkable overexpression of several types of MERVs (e.g., the IAP element) and neighbor genes from MERVs in mouse embryonic stem cells (ESCs) and initial events during embryogenesis [213,214,215].

MERV-L expression is high in embryonic stem cells [191]. The TF Zscan4c, aided by its zinc finger domains, acts as an inducer of the embryonic genes in 2-cell/4-cell embryos as well as MERV-L LTR (MT2) [216]. In addition to acting as a trigger for MT2, Zscan4c drives cellular regulation by stimulating the neighboring genes of MT2 (2C genes) in two-cell/four-cell embryos [216]. Zscan4c binds to the GLTSCR1/like-containing BAF complex (GBAF), a chromatin remodeling complex, via its SCAN domains to efficiently initiate MT2 functions, as determined by ChIP analysis [216]. On the other hand, MERV-L LTR is activated by DUX in the 2C-embryo-like cells [140,141,217,218,219]. In addition, p53 is required for the DUX expression in FSHD cells [220]. The DUX expression is repressed by the recruitment of Nucleolin/Kap1 with LINE1 RNA, thus the LINE1-Nucleolin complex indirectly represses the MERV-L LTR [221]. Moreover, PIM3 represses MERV-L via the hypo-phosphorylated HDAC4/5 [222]. ERVs may also exhibit a noticeable diversity in their expression models. In particular, these differences in ERV expression are observed at the different stages of embryogenesis, and the expression patterns are diverse among species. A vivid illustration of this can be seen in humans, where LTR14B, an affiliate of the HERV-K family, and HERV-L (MLT2A1) are augmented during the two-cell phase and in the four-cell/eight-cell embryo, respectively [216,223,224].

MERV-L LTRs are key for the expression of several cellular genes during zygotic genome activation (ZGA) and blastocyte-phase gene silencing [225]. Mutations in a lysine-specific demethylase (LSD1/KDM1A) boost the expression of ZGA-associated LTR, leading to an increase in cell propagation capabilities [225]. In ESCs, LSD1/KDM1A regulates the addition of methyl and acetyl groups during histone methylation of LTR sequences, resulting in MERV silencing. In contrast, MERV-L expression is high in ES and blastocyst-stage embryonic cells deficient in LSD1/KDM1A [225].

MERV-L expression is reported to largely depend on reduced expression-1 (Rex1), also known as Zfp42. Rex1/Zfp42 in murine ES cells attenuates MERV-L expression [226]. This mechanism of MERV-L deregulation is a constituent of the phenotypic primordial aberrations observed in Rex1/Zfp42-depleted mouse ESCs [226]. Rex1/Zfp42 is expressed in a wide variety of cells; it is expressed in pluripotent cells (predominantly in undifferentiated ES cells), multipotent adult progenitor cells, amniotic fluid (of testis germ cells, ICM), and derivatives of the trophectoderm from mouse embryos [227,228,229]. Moreover, Rex1/Zfp42 monitors MERV-L expression at the preimplantation developmental stage. Numerous regions responsible for TF binding have been identified in murine LTRs (e.g., RLTR9B2, RLTR9D, and RLTR9E) [230]. In particular, Esrrb, Klf4, and Sox2 enhance the expression of RLTR9B2, RLTR9D, and RLTR9E, respectively [230]. Esrrb, Klf4, and Sox2 are well-known reprogramming factors and master regulators of pluripotency (Table 2). In summary, the regulation of MERV-L by repression factors (Rex1/Zfp42 and LSD1/KDM1A) and activation factors (Esrrb, Klf4, and Sox2) may be crucial for reprogramming, stable pluripotency, and/or blastocyst development.

Studies on trophoblast stem cells (TSCs) from rats and mice have added to the understanding of the molecular mechanisms underlying placental development [231]. RLTR13D5, a member of the ERV family, generates hundreds of enhancers in mice that communicate with Elf5-core proteins, Eomes, and Cdx2, serving as the foundation of the TSC regulatory complex. Histone H3 lysine 4 monomethylation (H3K4me1) and histone H3 lysine 27 acetylation (H3K27ac) are two examples of these mediators [231]. In addition to RLTR13D5, exceedingly high levels of Eomes, Elf5, and Cdx2 were detected in RLTR13B4. These findings suggest that RLTR13D5 may coordinate gene expression in the rat placenta in the presence of Eomes, Elf5, and Cdx2 [231].

### 4.2. TFs Associated with LTR Activation of PERVs

PERVs have been inserted into the genome of pigs [249]. Owing to the presence of human PERV-A receptors 1 and 2 (huPAR1 and huPAR1, respectively) [250], human cells are susceptible to many types of PERVs, such as PERV-A, PERV-B, and PERV-A/C [249,251,252,253]. Therefore, xenotransplant recipients from pig donors with PERV expression cannot ignore the risk of PERV infection [232]. PERVs adapted to human cells do not produce mutations in *env* but alter the length of the LTRs [232]. For example, nuclear factor Y (NF-Y)-binding motif (CCAAT box) has been identified within PERV LTRs in 293T cells [232,254,255]. Similarly, LTR3, a member of the PERV-A family, encodes the transcription activators Nkx2-2 and Elk-1 [256]. According to the TRANSFAC database, several TFS (Sox5, Ets-1, Evi1, GATA, v-Myb, and CEBP; Table 2) have been identified within the PERV-C LTR [233]. Although studies have investigated the mechanism of transcriptional activation of PERVs after adaptation to human cells, the pathogenicity of PERVs that are activated in human cells remains unclear.

### 4.3. TFs Associated with LTR Activation of SERVs

SERVs exist as two types: Cer-SERV-1 and Cer-SERV-2 [235]. The sequences within the LTR of Cer-SERV-1 are approximately 484 nt in length and encode five to eight TF-binding motifs (CREB, CDP, E2F, AREB6, FoxD3, FoxJ2, and Brn-2), shown in Table 2 [235]. As FoxD3 and FoxJ2 are expressed in embryonic stem cells and during early embryonic development, respectively [234,257], Cer-SERV-1 may also be expressed in embryonic stem cell and during early embryonic development. Cer-SERV-2 encodes other TFs (AREB6, COMP1, CREB, NF-1, RFX1, Pax6, and v-Myb) than those encoded by Cer-SERV-1 [235]. Therefore, the transcription of Cer-SERV-2 may be regulated by other elements in different cells. The monkey-specific LTR (MacERV6-LTR1a) is briefly activated in blastocysts before implantation and is silenced after implantation. MacERV6-LTR1a recruits Esrrb, but not Klf4, Oct4, Sox2, SMAD3, or HNF4A, to activate transcription [236]. Importantly, similar to other ERVs, SERVs are expressed early during development.

### 4.4. TFs Associated with LTR Activation of Bovine ERVs (BoERVs)

Genomic expression of transposable elements (TEs) depends largely on their translocation [258]. The LTR regulatory element of the TEs plays a vital role in this process [4]. Genomic studies have shown that 27% of the bovine genome consists of TEs [259]. These TEs contribute to the genomic structure and evolution of cattle [259]. The insertion of TEs may result in neighboring gene modulation and chromosomal duplications [237]. However, the expression of BoERVs varies according to developmental stage, organ, and tissue type [237]. For example, BoERVs located on chromosomes 2 and 4 are highly expressed in endocrine glands [237]. Gene ontology analysis revealed that the LTRs of BoERV16, BoERV3, and BoERV9 contain a binding motif for the TF retinoid-X receptor alpha (Rxra), which upregulates BoERV expression in the thyroid [237]. In contrast, the ruminant-specific TEs MER41_BT and Bov-A2 are mainly expressed in bovine eight-cell embryos during development [240]. ATAC-seq analysis revealed that pluripotency factors/TFs, namely Oct4, NFY, Klf4, OTX2, TEAD, and STAT1, bind to MER41_BT and STAT1 [238], while POLR2A binds to Bov-A2 [239], thereby upregulating the expression of BoERVs. The basic principle of retroviral replication is binding of TFs to the U3 region of retroviral LTR promoter [260]. Bovine retrovirus BoRV CH15 is thought to be endogenous and causes neural disease upon activation. In silico analysis revealed the presence of binding sites for a putative TF nuclear factor-1 (NF-1) in its LTRs; NF-1 likely activates BoRV CH15 expression, damaging the brain neurons and causing cattle encephalitis [240]. Thus, further studies on TFs might be key to understanding its incentive as well as maintaining bovine health.

### 4.5. TFs Associated with LTR Activation of Feline Endogenous Leukemia Virus (enFeRVs)

Endogenous feline leukemia virus (enFeLV), which is dispersed throughout the cat genome [261], has a full-length pro-viral genome [262]. The enFeLV transcript is expressed in almost all cats [263]. Active enFeLV accelerates the diseases progression, for instance, autoimmune disease [264]. Therefore, the activation of enFeLV is concerning in domestic animal health, especially in cats. The viral load of enFeLV is elevated in the FeLV-infected cats [265]. ERV-DCs, which is the latest characterized class of enFeLVs [266], express a couple of truncated env proteins known as Refrex-1 [93]. Refrex-1 has a strong inhibitory effect on the activation or reemergence of the ERV-DCs [93] (Figure 1E). In addition, enFeLV microRNA is highly expressed in PBMCs and may inhibit the exogenous FeLV replication in PBMCs [92]. On the other hand, the transcription factors responsible for the LTR activation of enFeLV are not well revealed yet. The higher CpG methylation in the ERV-DC10 LTR reduced the expression of ERV-DC significantly [266], hence, CpG methylation controls the LTR activity of feline ERVs. The ERV-DC LTRs have been classified based on the differences in nucleotide sequences [266], and these nucleotide substitutions might influence the basal promoter activity of LTRs [266]. Further study on transcription faction is essential to unveil the physiological significances of interference between enFeLV and exogenous FeLV.

### 4.6. TFs Associated with LTR Activation of ZFERVs

Expressed-Zebrafish-Retroelement group 1 (EZR1) comprises approximately 8% of the zebrafish genome [267]. EZR1 potentially encodes LTR, *integrase,* and *env* genes but not structural genes such as *gag* [241]. EZR1 transcripts are detected in several adult tissues, such as the heart, and whole embryos [241]. Several TFs (TCF-11, Nkx-2.5, GATA-1, MZF-1, Ikaros-2) (Table 2) are candidate elements for EZR1 transcription; in particular, the zebrafish Rel family protein homologs interact with a putative NF-κB-binding motif within the LTRs [241]. Since EZR1 is expressed in a wide range of cell types via many potential TFs, the silencing mechanisms of EZR1, such as Ziwi [267], may be important for understanding the evolution of organisms.

### 4.7. TFs Associated with LTR Activation of Yeast Retrotransposons

In *Saccharomyces cerevisiae*, yeast retrotransposons Ty1–Ty5 have been discovered, with Ty1/Copia being the most abundant [268]. Ty1 elements contain two ORFs: TYA (*gag*) and TYB (*pol*) [269]. Forty years ago, Ty1 was discovered as an LTR-retrotransposon that transposes through RNA to move its genes [270]. Subsequently, several LTR-retrotransposons that transpose via RNA have been discovered [107,271,272,273,274].

Transcription of Ty1 is regulated by several TFs (Gcr1, Ste12, Tec1, Mcm1, Tea1/Ibf1, Rap1, Gcn4, Mot3, and Tye7), shown in Table 2, and chromatin-remodeling complexes (Swi/Snf, SAGA, and ISWI) [244,246,247,275,276,277,278,279,280,281,282,283]. In haploid cells, transcription of Ty1 requires Ste12 and Tec1 that cooperatively bind to filamentation- and invasion-responsive element (FRE) sites located near the TATA box [242,243,275].

Furthermore, TF Mot3, a type of zinc finger protein, binds to the Ty912δ LTR. Ty912δ, a TE from Ty1, is integrated into the HIS4 promoter [284]. Mot3 regulates Ty912δ expression as either a repressor or activator [244]. Deletion of genes encoding the Gcn5 protein within the SAGA complex severely decreases Ty1 transcription [278]. Other Gcn genes also exert mild effects on Ty1 transcription [245,281,283,285]. Mcm1 interacts with the Tyl block II motif [246]. Generally, Mcm1 forms complexes with various TFs [286]. However, the Mcm1-binding motif is far from the binding motifs of other factors that interact with Mcm1, suggesting that Mcm1 may activate Ty1 transcription via an out-of-the-ordinary pathway [246]. The binding sites for the TF Rap1 are within the internal regulatory region of Ty1 elements. Rap1 plays an important regulatory role in the expression of Ty1 and Ty1-mediated adjacent genes. RAP1 strongly activates Ty1 transcription by forming a complex with Mcm1 [287]. The binding site of the TF Tea1 is located near the binding sites of Rap1 and Mcm1 [247], suggesting that the Tea1, Rap1, and Mcm1 complex regulates Ty1 transcription.

### 4.8. TFs Associated with the Drosophila (Gypsy)

In Drosophila, Ty3/gypsy is an LTR-containing TE on the X chromosome [288]. Motif 1 binding protein (M1BP) is a gypsy-activating TF ubiquitously expressed in *Drosophila* cells [248]. ChIP-seq revealed that M1BP interacts with the centrosomal protein (CP190) and activates motif 1-dependent transcription, followed by gypsy insulator activity [248].

## 5. Conclusions

The eukaryotic genome contains vestiges of ERVs, particularly at ~400,000 loci in humans [289]. Despite the loss of their innate virulence, ERVs possess full-length ORFs and can participate in cellular functions [4,115]. Indeed, ERVs are associated with cellular functions that may be beneficial or lead to disease progression in the host cells [58,59]. This raises the question of how they are activated in the genome. TFs are the prime elements that activate ERVs owing to the presence of the classical binding motif on their LTRs [94].

This review summarized the TFs that activate ERVs in various species and discussed their corresponding physiological roles. In cancer cells, Sox2, Oct4, and Nanog activate HERV-K 5Hs, leading to neurological illnesses. Also, c-Myc activates HERV-K, thereby facilitating conditions such as atypical teratoid rhabdoid tumors. Some TFs (such as YYI) possess both HERV activator and repressor activities. HERV-L and HERV-E, which are activated by the TFs DUX4 and HIF, respectively, are associated with cancer. On the other hand, DUX/DUX4 is a crucial regulator of ZGA gene expression and important for forming cultured two-cell-like cells. Alternatively, HERV-W and RLTR13D5 *env* protein (Syncytin-1) are known to participate in placental development in both humans and mice [183,231]. Human TFs, c-Myb, murine Cdx2, Eomes, and Elf5 are responsible for activating ERVs during placental formation. The Solo-LTRs are actively involved in cellular functions. For instance, upon IFN-γ stimulation, the solo-LTR12F switches on the full-length HERV-K102 in patients with cutaneous leishmaniasis [124], and IFN-γ and IFN-α together significantly activate HERV-K18 in T cells of patients with type-1 diabetes [126]. A similar phenomenon was observed for the upregulation of the HERV-H solo-LTR on chromosome 17q21 by Sp1, Oct4, Nanog, and LBP9. Prominent inducers of MERVs and MacERV6-LTR1 include Oct4, Sox2, and NANOG. In addition, Zscan4c, Gata4, Cdx2, and Elf5 activate MERVs. Notably, the most explored TF that triggers MacERV6-LTR1 is KLF4. The gypsy LTR in Drosophila is activated by M1BP and counteracted by the su protein. The zebrafish ERV EZR1 is primarily induced by Oct-1 and NF-κB. Moreover, yeast endogenous retrovirus (Ty1) is involved in cellular metabolism and is activated by Gcr1/4, Ste12, Tec1, Mcm1, Tea1/Ibf1, Rap1, Gcn1/4/5, Mot3, and Tye7 [275].

The fact that ERVs continue to be inscribed into the genome through the long process of biological evolution suggests that ERVs are not just fossils. In most animal species, ERVs are activated by TFs that are expressed early in development. Attractively, transcription factors expressed in early development, such as DUX, Sox2, and Oct4, are commonly used throughout the process of biological evolution. Considering that ERVs persist in utilizing these TFs during the long coexistence period of the host and virus confirms that ERVs may play a crucial role in early development. As the expression of these TFs is associated with various diseases, the causal relationship between ERVs and diseases needs to be carefully investigated in the future.

## Figures and Tables

**Table 1:** List of human endogenous retroviruses, their transcription factors, chromosomal location, and associated functions. HERV: human endogenous retrovirus; ERV: endogenous retrovirus; DUX: double homeobox 4; HIF: hypoxia-inducible transcription factor; NF-κB: nuclear factor-κB; YY1; Yin Yang 1; chr: chromosome; AT/RT: atypical teratoid rhabdoid tumor; ALS: amyotrophic lateral sclerosis; IFN: interferon; SLE: systemic lupus erythematosus.

Species	Viruses	Transcription Factors	Chromosome	Disease/Function	Reference
**Human**	HERV-K5Hs, 5A, 5B	Sox2, Oct4, and Nanog	chr5q33.3	Neurodegenerative disease	[107,108]
	HERV-K and HERV-L	DUX4	chr17q and chr7p22	FSHD	[115]
	HERV-K5Hs, and HERV-1	HIF		Kidney cancer	[116]
	HERV-K5Hs	c-Myc	chr7p22.1a and chr7p22.1b	AT/RT	[95]
	HERV-K5Hs	NF-κB and IRF1		ALS	[117]
	HERV-K5Hs	PR/Estradiol		Breast cancer	[118]
	HERV-K5Hs and HERV-E	Sp1 and Sp3		Skin cancer	[103]
	HERV-K5Hs	TDP-43		ALS	[119]
	HERV-K5Hs, 5A, 5B	p53, p60, and p65		Leukemia, ovarian, and colorectal cancers	[101,104]
	HERV-K5Hs	YY1		Cancers	[120,121]
	HERV-K	NF-AT		HIV associated malignancy	[122]
	HERV-K	MITF-M	chr1	Melanoma	[123]
	HERV-K	STAT-1 and IRF1		Inflammatory disease and IFN-γ signaling	[124]
	HERV-K	USF-1		Wound repair regulation	[108]
	HERV-K	Tax		Associated with opportunistic infection	[125]
	HERV-K	Tat		Associated with opportunistic infection	[122]
	HERV-K18	IFN-α	chr1q21.2-1q22	Type-1 diabetes	[126]
	HERV-K102	IFN- γ		Leishmaniasis	[124]
	HERV-L	HNF-1		Colon cancer	[79]
	HERV-W	c-Myb/HOXA5	chr7q21.2	Tumor progression	[127]
	HERV-W	OCT-1		Cell abnormalities	[128]
	HERV-W	Sp1 and Sp3		Lung fibroblasts	[129]
	HERV-W	GCM-a	chr7q21-7q22	Placental formation	[130]
	HERV-E	HIF-2α HIFs, HIF-1α, HIF-2α, and HIF-1β		Kidney cancer	[131]
	HERV-E	*NAFT1*	chr19p12	SLE	[132]
	HERV-H	Sp1, GC box, and TATA box		Breast cancer	[133,134,135,136]
	HERV-H	LBP9, Oct4, Nanog, and Klf4		Chromosome duplication	[137]
	HERV-T	GATA4, and FOXA2		Endoderm specification	[138]
	ERV-9	GATA-2, NF-Y, and MZF1		Chromatin remodeling	[139]

**Table 2:** List of species-specific endogenous retroviruses excluding humans, their transcription factors, chromosomal locations, and the associated functions. MERV: murine endogenous retrovirus; LTR: long terminal repeat; PERV: porcine endogenous retrovirus; SERV: simian endogenous retrovirus; EZR: expressed-zebrafish-retroelement group type 1; ZFERV: zebrafish endogenous retrovirus; TRIM28: tripartite motif-containing protein 28; NF-κB: nuclear factor-κB; chr: chromosome.

Species	Viruses	Transcription Factors	Chromosome	Disease/Function	Reference
**Mouse**	MERV-L LTR1	Zscan4c		Impair the embryonic development	[216]
	MERV-L LTR	DUX			[140]
	MERV-L RLTR9B2, RLTR9D, and RLTR9E	Esrrb, Klf4, and Sox2		Abnormality in cell pluripotency	[230]
	MERV-L LTR1	KDM1A		Impair the embryonic development	[225]
	MERV-RLTR13D5	Cdx2, Eomes, and Elf5		Regulate the placental development	[231]
**Pig**	PERV-A, PERV-B, and PERV-C LTR	NF-AT, Oct-1, Ets-1, v-myb, HFH-3, NF-1, AP-1, NF-Y, and AP-1C		Impair the embryonic development	[232,233]
**Monkey**	Cer-SERV-1 LTRs	AREB6, Brn-2, CDP, CREB, E2F, FoxD3, and FoxJ2		Embryonic development	[234]
	Cer-SERV-2 LTRs	AREB6, COMP1, CREB, NF-1, RFX1, Pax6, and v-Myb		Embryonic development	[235]
	MacERV6-LTR1	ESRRB		Embryonic development	[236]
**Bovine**	BoERV3, BoERV9, and BoERV16	Rxra	chr2 and chr4	Endocrine function	[237]
	MER41_BT	Oct4, NFY, Klf4, OTX2, TEAD, and STAT1			[238]
	Bov-A2	POLR2A			[239]
	BoRV CH15	NF-1		Cattle encephalitis	[240]
**Zebra Fish**	EZR1	NF-κB	chr4 and chr5	Cardiac malfunction	[241]
	EZR1	TCF-11, Nkx-2.5, GATA-1, MZF-1, and Ikaros-2		Lymphocyte maturation	[241]
**Yeast**	Ty1 LTR	Ste12 and Tec1		Cell cycle regulation	[242,243]
	Ty912d LTR	Mot3		Gene activation or repression	[244]
	Ty1 LTR	Gcn4, and Gcr1		Glucose metabolism	[245]
	Ty1 LTR	Mcm1		Cellular metabolism	[246]
	Ty1 LTR	Tea1 and Rap1		Cellular metabolism	[247]
**Drosophila**	Ty3/Gypsy	M1BP	chrX		[248]

## Data Availability

No new data were created.

## References

[1] Löwer R., Löwer J., Tondera-Koch C., Kurth R. (1993). A general method for the identification of transcribed retrovirus sequences (R-U5 PCR) reveals the expression of the human endogenous retrovirus loci HERV-H and HERV-K in teratocarcinoma cells. Virology.

[2] Skirmuntt E.C., Escalera-Zamudio M., Teeling E.C., Smith A., Katzourakis A. (2020). The Potential Role of Endogenous Viral Elements in the Evolution of Bats as Reservoirs for Zoonotic Viruses. Annu. Rev. Virol..

[3] Aiewsakun P., Katzourakis A. (2015). Endogenous viruses: Connecting recent and ancient viral evolution. Virology.

[4] Jern P., Coffin J.M. (2008). Effects of retroviruses on host genome function. Annu. Rev. Genet..

[5] Ono M., Yasunaga T., Miyata T., Ushikubo H. (1986). Nucleotide sequence of human endogenous retrovirus genome related to the mouse mammary tumor virus genome. J. Virol..

[6] Boeke J.D., Stoye J.P., Coffin J.M., Hughes S.H., Varmus H.E. (1997). Retrotransposons, Endogenous Retroviruses, and the Evolution of Retroelements. Retroviruses.

[7] Bieda K., Hoffmann A., Boller K. (2001). Phenotypic heterogeneity of human endogenous retrovirus particles produced by teratocarcinoma cell lines. J. Gen. Virol..

[8] Bronson D.L., Fraley E.E., Fogh J., Kalter S.S. (1979). Induction of retrovirus particles in human testicular tumor (Tera-1) cell cultures: An electron microscopic study. JNCI J. Natl. Cancer Inst..

[9] Boller K., König H., Sauter M., Mueller-Lantzsch N., Löwer R., Löwer J., Kurth R. (1993). Evidence that HERV-K is the endogenous retrovirus sequence that codes for the human teratocarcinoma-derived retrovirus HTDV. Virology.

[10] Subramanian R.P., Wildschutte J.H., Russo C., Coffin J.M. (2011). Identification, characterization, and comparative genomic distribution of the HERV-K (HML-2) group of human endogenous retroviruses. Retrovirology.

[11] Srinivasachar Badarinarayan S., Sauter D. (2021). Switching Sides: How Endogenous Retroviruses Protect Us from Viral Infections. J. Virol..

[12] Boller K., Schönfeld K., Lischer S., Fischer N., Hoffmann A., Kurth R., Tönjes R.R. (2008). Human endogenous retrovirus HERV-K113 is capable of producing intact viral particles. J. Gen. Virol..

[13] Wildschutte J.H., Williams Z.H., Montesion M., Subramanian R.P., Kidd J.M., Coffin J.M. (2016). Discovery of unfixed endogenous retrovirus insertions in diverse human populations. Proc. Natl. Acad. Sci. USA.

[14] Turner G., Barbulescu M., Su M., Jensen-Seaman M.I., Kidd K.K., Lenz J. (2001). Insertional polymorphisms of full-length endogenous retroviruses in humans. Curr. Biol..

[15] Belshaw R., Pereira V., Katzourakis A., Talbot G., Pačes J., Burt A., Tristem M. (2004). Long-term reinfection of the human genome by endogenous retroviruses. Proc. Natl. Acad. Sci. USA.

[16] Belshaw R., Dawson A.L.A., Woolven-Allen J., Redding J., Burt A., Tristem M. (2005). Genomewide screening reveals high levels of insertional polymorphism in the human endogenous retrovirus family HERV-K(HML2): Implications for present-day activity. J. Virol..

[17] Durnaoglu S., Lee S.-K., Ahnn J. (2021). Human Endogenous Retroviruses as Gene Expression Regulators: Insights from Animal Models into Human Diseases. Mol. Cells.

[18] Bock M., Stoye J.P. (2000). Endogenous retroviruses and the human germline. Curr. Opin. Genet. Dev..

[19] McPherson J.D., Marra M., Hillier L., Waterston R.H., Chinwalla A., Wallis J., Sekhon M., Wylie K., Mardis E.R., Wilson R.K. (2001). A physical map of the human genome. Nature.

[20] Bannert N., Kurth R. (2004). Retroelements and the human genome: New perspectives on an old relation. Proc. Natl. Acad. Sci. USA.

[21] Lander E.S., Linton L.M., Birren B., Nusbaum C., Zody M.C., Baldwin J., Devon K., Dewar K., Doyle M., FitzHugh W. (2001). Initial sequencing and analysis of the human genome. Nature.

[22] Venter J.C., Adams M.D., Myers E.W., Li P.W., Mural R.J., Sutton G.G., Smith H.O., Yandell M., Evans C.A., Holt R.A. (2001). The sequence of the human genome. Science.

[23] Grow E.J., Flynn R.A., Chavez S.L., Bayless N.L., Mark W., Wesche D.J., Lance M., Ware C.B., Blish C.A., Chang H.Y. (2015). Intrinsic retroviral reactivation in human preimplantation embryos and pluripotent cells. Nature.

[24] Kato N., Pfeifer-Ohlsson S., Kato M., Larsson E., Rydnert J., Ohlsson R., Cohen M. (1987). Tissue-specific expression of human provirus ERV3 mRNA in human placenta: Two of the three ERV3 mRNAs contain human cellular sequences. J. Virol..

[25] Kjellman C., Sjögren H.-O., Salford L.G., Widegren B. (1999). HERV-F(XA34) is a full-length human endogenous retrovirus expressed in placental and fetal tissues. Gene.

[26] Mallet F., Bouton O., Prudhomme S., Cheynet V., Oriol G., Bonnaud B., Lucotte G., Duret L., Mandrand B. (2004). The endogenous retroviral locus ERVWE1 is a bona fide gene involved in hominoid placental physiology. Proc. Natl. Acad. Sci. USA.

[27] Prudhomme S., Oriol G., Mallet F. (2004). A retroviral promoter and a cellular enhancer define a bipartite element which controls *env* ervwe1 placental expression. J. Virol..

[28] Frank O., Verbeke C., Schwarz N., Mayer J., Fabarius A., Hehlmann R., Leib-Mösch C., Seifarth W. (2008). Variable transcriptional activity of endogenous retroviruses in human breast cancer. J. Virol..

[29] Ono M., Kawakami M., Ushikubo H. (1987). Stimulation of expression of the human endogenous retrovirus genome by female steroid hormones in human breast cancer cell line T47D. J. Virol..

[30] Wang-Johanning F., Frost A.R., Jian B., Epp L., Lu D.W., Johanning G.L. (2003). Quantitation of HERV-K env gene expression and splicing in human breast cancer. Oncogene.

[31] Wang-Johanning F., Frost A.R., Johanning G.L., Khazaeli M.B., LoBuglio A.F., Shaw D.R., Strong T.V. (2001). Expression of human endogenous retrovirus k envelope transcripts in human breast cancer. Clin. Cancer Res..

[32] Willer A., Saußele S., Gimbel W., Zeifarth W., Kister P., Leib-Mösch C., Hehlmann R. (1997). Two groups of endogenous MMTV related retroviral env transcripts expressed in human tissues. Virus Genes.

[33] Herbst H., Sauter M., Kühler-Obbarius C., Löning T., Mueller-Lantzsch N. (1998). Human endogenous retrovirus (HERV)-K transcripts in germ cell and trophoblastic tumours. APMIS.

[34] Sauter M., Roemer K., Best B., Afting M., Schommer S., Seitz G., Hartmann M., Mueller-Lantzsch N. (1996). Specificity of antibodies directed against Env protein of human endogenous retroviruses in patients with germ cell tumors. Cancer Res..

[35] Flockerzi A., Ruggieri A., Frank O., Sauter M., Maldener E., Kopper B., Wullich B., Seifarth W., Müller-Lantzsch N., Leib-Mösch C. (2008). Expression patterns of transcribed human endogenous retrovirus HERV-K(HML-2) loci in human tissues and the need for a HERV Transcriptome Project. BMC Genom..

[36] Ruprecht K., Ferreira H., Flockerzi A., Wahl S., Sauter M., Mayer J., Mueller-Lantzsch N. (2008). Human endogenous retrovirus family HERV-K(HML-2) RNA transcripts are selectively packaged into retroviral particles produced by the human germ cell tumor line Tera-1 and originate mainly from a provirus on chromosome 22q11.21. J. Virol..

[37] Büscher K., Hahn S., Hofmann M., Trefzer U., Özel M., Sterry W., Löwer J., Löwer R., Kurth R., Denner J. (2006). Expression of the human endogenous retrovirus-K transmembrane envelope, Rec and Np9 proteins in melanomas and melanoma cell lines. Melanoma Res..

[38] Hahn S., Ugurel S., Hanschmann K.-M., Strobel H., Tondera C., Schadendorf D., Löwer J., Löwer R. (2008). Serological response to human endogenous retrovirus K in melanoma patients correlates with survival probability. AIDS Res. Hum. Retroviruses.

[39] Mangeney M., Pothlichet J., Renard M., Ducos B., Heidmann T. (2005). Endogenous retrovirus expression is required for murine melanoma tumor growth in vivo. Cancer Res..

[40] Muster T., Waltenberger A., Grassauer A., Hirschl S., Caucig P., Romirer I., Födinger D., Seppele H., Schanab O., Magin-Lachmann C. (2003). An endogenous retrovirus derived from human melanoma cells. Cancer Res..

[41] Hu L., Hornung D., Kurek R., Östman H., Blomberg J., Bergqvist A. (2006). Expression of human endogenous gammaretroviral sequences in endometriosis and ovarian cancer. AIDS Res. Hum. Retroviruses.

[42] Wang-Johanning F., Liu J., Rycaj K., Huang M., Tsai K., Rosen D.G., Chen D., Lu D.W., Barnhart K.F., Johanning G.L. (2007). Expression of multiple human endogenous retrovirus surface envelope proteins in ovarian cancer. Int. J. Cancer.

[43] Iwabuchi H., Kakihara T., Kobayashi T., Imai C., Tanaka A., Uchiyama M., Fukuda T. (2004). A Gene homologous to human endogenous retrovirus overexpressed in childhood acute lymphoblastic leukemia. Leuk. Lymphoma.

[44] Contreras-Galindo R., Kaplan M.H., Leissner P., Verjat T., Ferlenghi I., Bagnoli F., Giusti F., Dosik M.H., Hayes D.F., Gitlin S.D. (2008). Human endogenous retrovirus K (HML-2) elements in the plasma of people with lymphoma and breast cancer. J. Virol..

[45] Ng K.W., Boumelha J., Enfield K.S.S., Almagro J., Cha H., Pich O., Karasaki T., Moore D.A., Salgado R., Sivakumar M. (2023). Antibodies against endogenous retroviruses promote lung cancer immunotherapy. Nature.

[46] Dickerson F., Rubalcaba E., Viscidi R., Yang S., Stallings C., Sullens A., Origoni A., Leister F., Yolken R. (2008). Polymorphisms in human endogenous retrovirus K-18 and risk of type 2 diabetes in individuals with schizophrenia. Schizophr. Res..

[47] Frank O., Giehl M., Zheng C., Hehlmann R., Leib-Mösch C., Seifarth W. (2005). Human endogenous retrovirus expression profiles in samples from brains of patients with schizophrenia and bipolar disorders. J. Virol..

[48] Huang W.-J., Liu Z.-C., Wei W., Wang G.-H., Wu J.-G., Zhu F. (2006). Human endogenous retroviral pol RNA and protein detected and identified in the blood of individuals with schizophrenia. Schizophr. Res..

[49] Karlsson H., Bachmann S., Schröder J., McArthur J., Torrey E.F., Yolken R.H. (2001). Retroviral RNA identified in the cerebrospinal fluids and brains of individuals with schizophrenia. Proc. Natl. Acad. Sci. USA.

[50] Sicat J., Sutkowski N., Huber B.T. (2005). Expression of human endogenous retrovirus HERV-K18 superantigen is elevated in juvenile rheumatoid arthritis. J. Rheumatol..

[51] Ehlhardt S., Seifert M., Schneider J., Ojak A., Zang K.D., Mehraein Y. (2006). Human endogenous retrovirus HERV-K(HML-2) Rec expression and transcriptional activities in normal and rheumatoid arthritis synovia. J. Rheumatol..

[52] Reynier F., Verjat T., Turrel F., Imbert P.E., Marotte H., Mougin B., Miossec P. (2009). Increase in human endogenous retrovirus HERV-K (HML-2) viral load in active rheumatoid arthritis. Scand. J. Immunol..

[53] Freimanis G., Hooley P., Ejtehadi H.D., Ali H.A., Veitch A., Rylance P.B., Alawi A., Axford J., Nevill A., Murray P.G. (2010). A role for human endogenous retrovirus-K (HML-2) in rheumatoid arthritis: Investigating mechanisms of pathogenesis. Clin. Exp. Immunol..

[54] Guo C., Jeong H.-H., Hsieh Y.-C., Klein H.-U., Bennett D.A., De Jager P.L., Liu Z., Shulman J.M. (2018). Tau Activates Transposable Elements in Alzheimer’s Disease. Cell Rep..

[55] Douville R., Liu J., Rothstein J., Nath A. (2011). Identification of active loci of a human endogenous retrovirus in neurons of patients with amyotrophic lateral sclerosis. Ann. Neurol..

[56] Sakurai T., Nakagawa S., Bai H., Bai R., Kusama K., Ideta A., Aoyagi Y., Kaneko K., Iga K., Yasuda J. (2017). Novel endogenous retrovirus-derived transcript expressed in the bovine placenta is regulated by WNT signaling. Biochem. J..

[57] She J., Du M., Xu Z., Jin Y., Li Y., Zhang D., Tao C., Chen J., Wang J., Yang E. (2022). The landscape of hervRNAs transcribed from human endogenous retroviruses across human body sites. Genome Biol..

[58] Contreras-Galindo R., Kaplan M.H., Markovitz D.M., Lorenzo E., Yamamura Y. (2006). Detection of HERV-K(HML-2) viral rna in plasma of hiv type 1-infected individuals. AIDS Res. Hum. Retroviruses.

[59] Contreras-Galindo R., López P., Vélez R., Yamamura Y. (2007). HIV-1 infection increases the expression of human endogenous retroviruses type K (HERV-K) in vitro. AIDS Res. Hum. Retroviruses.

[60] Dai L., Del Valle L., Miley W., Whitby D., Ochoa A.C., Flemington E.K., Qin Z. (2018). Transactivation of human endogenous retrovirus K (HERV-K) by KSHV promotes Kaposi’s sarcoma development. Oncogene.

[61] Hughes J.F., Coffin J.M. (2004). Human endogenous retrovirus K solo-LTR formation and insertional polymorphisms: Implications for human and viral evolution. Proc. Natl. Acad. Sci. USA.

[62] Fuentes D.R., Swigut T., Wysocka J. (2018). Systematic perturbation of retroviral LTRs reveals widespread long-range effects on human gene regulation. Elife.

[63] Oricchio E., Sciamanna I., Beraldi R., Tolstonog G.V., Schumann G.G., Spadafora C. (2007). Distinct roles for LINE-1 and HERV-K retroelements in cell proliferation, differentiation and tumor progression. Oncogene.

[64] Pisano M.P., Grandi N., Cadeddu M., Blomberg J., Tramontano E. (2019). Comprehensive Characterization of the Human Endogenous Retrovirus HERV-K(HML-6) Group: Overview of Structure, Phylogeny, and Contribution to the Human Genome. J. Virol..

[65] Shin W., Mun S., Han K. (2023). Human Endogenous Retrovirus-K (HML-2)-Related Genetic Variation: Human Genome Diversity and Disease. Genes.

[66] Antony J.M., Deslauriers A.M., Bhat R.K., Ellestad K.K., Power C. (2011). Human endogenous retroviruses and multiple sclerosis: Innocent bystanders or disease determinants?. Biochim. Biophys. Acta.

[67] Balada E., Ordi-Ros J., Vilardell-Tarrés M. (2009). Molecular mechanisms mediated by human endogenous retroviruses (HERVs) in autoimmunity. Rev. Med. Virol..

[68] Gröger V., Emmer A., Staege M.S., Cynis H. (2021). Endogenous Retroviruses in Nervous System Disorders. Pharmaceuticals.

[69] Liu X., Liu Z., Wu Z., Ren J., Fan Y., Sun L., Cao G., Niu Y., Zhang B., Ji Q. (2023). Resurrection of endogenous retroviruses during aging reinforces senescence. Cell.

[70] Liu S., Heumüller S.-E., Hossinger A., Müller S.A., Buravlova O., Lichtenthaler S.F., Denner P., Vorberg I.M. (2023). Reactivated endogenous retroviruses promote protein aggregate spreading. Nat. Commun..

[71] Levinson B., Khoury G., Woude G.V., Gruss P. (1982). Activation of SV40 genome by 72-base pair tandem repeats of Moloney sarcoma virus. Nature.

[72] Yaniv M. (1982). Enhancing elements for activation of eukaryotic promoters. Nature.

[73] Domansky A.N., Kopantzev E.P., Snezhkov E.V., Lebedev Y.B., Leib-Mosch C., Sverdlov E.D. (2000). Solitary HERV-K LTRs possess bi-directional promoter activity and contain a negative regulatory element in the U5 region. FEBS Lett..

[74] Yu H.-L., Zhao Z.-K., Zhu F. (2013). The role of human endogenous retroviral long terminal repeat sequences in human cancer (Review). Int. J. Mol. Med..

[75] Samuelson L.C., Wiebauer K., Snow C.M., Meisler M.H. (1990). Retroviral and pseudogene insertion sites reveal the lineage of human salivary and pancreatic amylase genes from a single gene during primate evolution. Mol. Cell. Biol..

[76] Medstrand P., Landry J.R., Mager D.L. (2001). Long terminal repeats are used as alternative promoters for the endothelin B receptor and apolipoprotein C-I genes in humans. J. Biol. Chem..

[77] Ruda V., Akopov S., Trubetskoy D., Manuylov N., Vetchinova A., Zavalova L., Nikolaev L., Sverdlov E. (2004). Tissue specificity of enhancer and promoter activities of a HERV-K(HML-2) LTR. Virus Res..

[78] Kapitonov V.V., Jurka J. (1999). The long terminal repeat of an endogenous retrovirus induces alternative splicing and encodes an additional carboxy-terminal sequence in the human leptin receptor. J. Mol. Evol..

[79] Dunn C.A., Medstrand P., Mager D.L. (2003). An endogenous retroviral long terminal repeat is the dominant promoter for human beta1,3-galactosyltransferase 5 in the colon. Proc. Natl. Acad. Sci. USA.

[80] Liu M., Eiden M.V. (2011). Role of human endogenous retroviral long terminal repeats (LTRs) in maintaining the integrity of the human germ line. Viruses.

[81] Landry J.R., Mager D.L. (2003). Functional analysis of the endogenous retroviral promoter of the human endothelin B receptor gene. J. Virol..

[82] Frank J.A., Singh M., Cullen H.B., Kirou R.A., Benkaddour-Boumzaouad M., Cortes J.L., Pérez J.G., Coyne C.B., Feschotte C. (2022). Evolution and antiviral activity of a human protein of retroviral origin. Science.

[83] Monde K., Contreras-Galindo R., Kaplan M.H., Markovitz D.M., Ono A. (2012). Human endogenous retrovirus K gag coassembles with HIV-1 gag and reduces the release efficiency and infectivity of HIV-1. J. Virol..

[84] Monde K., Terasawa H., Nakano Y., Soheilian F., Nagashima K., Maeda Y., Ono A. (2017). Molecular mechanisms by which HERV-K Gag interferes with HIV-1 Gag assembly and particle infectivity. Retrovirology.

[85] Malfavon-Borja R., Feschotte C. (2015). Fighting fire with fire: Endogenous retrovirus envelopes as restriction factors. J. Virol..

[86] Sugimoto J., Sugimoto M., Bernstein H., Jinno Y., Schust D. (2013). A novel human endogenous retroviral protein inhibits cell-cell fusion. Sci. Rep..

[87] Pastuzyn E.D., Day C.E., Kearns R.B., Kyrke-Smith M., Taibi A.V., McCormick J., Yoder N., Belnap D.M., Erlendsson S., Morado D.R. (2018). The Neuronal Gene Arc Encodes a Repurposed Retrotransposon Gag Protein that Mediates Intercellular RNA Transfer. Cell.

[88] Ashley J., Cordy B., Lucia D., Fradkin L.G., Budnik V., Thomson T. (2018). Retrovirus-like Gag Protein Arc1 Binds RNA and Traffics across Synaptic Boutons. Cell.

[89] Segel M., Lash B., Song J., Ladha A., Liu C.C., Jin X., Mekhedov S.L., Macrae R.K., Koonin E.V., Zhang F. (2021). Mammalian retrovirus-like protein PEG10 packages its own mRNA and can be pseudotyped for mRNA delivery. Science.

[90] Hartley J.W., Rowe W.P., Huebner R.J. (1970). Host-range restrictions of murine leukemia viruses in mouse embryo cell cultures. J. Virol..

[91] Steeves R., Lilly F. (1977). Interactions between host and viral genomes in mouse leukemia. Annu. Rev. Genet..

[92] Chiu E.S., McDonald C.A., VandeWoude S. (2021). Endogenous Feline Leukemia Virus (FeLV) siRNA Transcription May Interfere with Exogenous FeLV Infection. J. Virol..

[93] Ito J., Watanabe S., Hiratsuka T., Kuse K., Odahara Y., Ochi H., Kawamura M., Nishigaki K. (2013). Refrex-1, a soluble restriction factor against feline endogenous and exogenous retroviruses. J. Virol..

[94] Bannert N., Hofmann H., Block A., Hohn O. (2018). HERVs New Role in Cancer: From Accused Perpetrators to Cheerful Protectors. Front. Microbiol..

[95] Doucet-O’hare T.T., DiSanza B.L., DeMarino C., Atkinson A.L., Rosenblum J.S., Henderson L.J., Johnson K.R., Kowalak J., Garcia-Montojo M., Allen S.J. (2021). SMARCB1 deletion in atypical teratoid rhabdoid tumors results in human endogenous retrovirus K (HML-2) expression. Sci. Rep..

[96] Wang G., Wang F., Huang Q., Li Y., Liu Y., Wang Y. (2015). Understanding Transcription Factor Regulation by Integrating Gene Expression and DNase I Hypersensitive Sites. BioMed Res. Int..

[97] Maston G.A., Evans S.K., Green M.R. (2006). Transcriptional regulatory elements in the human genome. Annu. Rev. Genom. Hum. Genet..

[98] de Mendoza A., Sebé-Pedrós A., Šestak M.S., Matejčić M., Torruella G., Domazet-Lošo T., Ruiz-Trillo I. (2013). Transcription factor evolution in eukaryotes and the assembly of the regulatory toolkit in multicellular lineages. Proc. Natl. Acad. Sci. USA.

[99] Vaquerizas J.M., Kummerfeld S.K., Teichmann S.A., Luscombe N.M. (2009). A census of human transcription factors: Function, expression and evolution. Nat. Rev. Genet..

[100] Yesudhas D., Batool M., Anwar M.A., Panneerselvam S., Choi S. (2017). Proteins Recognizing DNA: Structural Uniqueness and Versatility of DNA-Binding Domains in Stem Cell Transcription Factors. Genes.

[101] Liu M., Jia L., Li H., Liu Y., Han J., Wang X., Li T., Li J., Zhang B., Zhai X. (2022). p53 Binding Sites in Long Terminal Repeat 5Hs (LTR5Hs) of Human Endogenous Retrovirus K Family (HML-2 Subgroup) Play Important Roles in the Regulation of LTR5Hs Transcriptional Activity. Microbiol. Spectr..

[102] Bhardwaj N., Montesion M., Roy F., Coffin J.M. (2015). Differential expression of HERV-K (HML-2) proviruses in cells and virions of the teratocarcinoma cell line Tera-1. Viruses.

[103] Fuchs N.V., Kraft M., Tondera C., Hanschmann K.M., Lower J., Lower R. (2011). Expression of the human endogenous retrovirus (HERV) group HML-2/HERV-K does not depend on canonical promoter elements but is regulated by transcription factors Sp1 and Sp3. J. Virol..

[104] Manghera M., Douville R.N. (2013). Endogenous retrovirus-K promoter: A landing strip for inflammatory transcription factors?. Retrovirology.

[105] Montesion M., Williams Z.H., Subramanian R.P., Kuperwasser C., Coffin J.M. (2018). Promoter expression of HERV-K (HML-2) provirus-derived sequences is related to LTR sequence variation and polymorphic transcription factor binding sites. Retrovirology.

[106] Ernst J., Kellis M. (2012). ChromHMM: Automating chromatin-state discovery and characterization. Nat. Methods.

[107] Monde K., Satou Y., Goto M., Uchiyama Y., Ito J., Kaitsuka T., Terasawa H., Monde N., Yamaga S., Matsusako T. (2022). Movements of Ancient Human Endogenous Retroviruses Detected in SOX2-Expressing Cells. J. Virol..

[108] Ito J., Sugimoto R., Nakaoka H., Yamada S., Kimura T., Hayano T., Inoue I. (2017). Systematic identification and characterization of regulatory elements derived from human endogenous retroviruses. PLOS Genet..

[109] Bareiss P.M., Paczulla A., Wang H., Schairer R., Wiehr S., Kohlhofer U., Rothfuss O.C., Fischer A., Perner S., Staebler A. (2013). SOX2 expression associates with stem cell state in human ovarian carcinoma. Cancer Res..

[110] Chen S., Xu Y., Chen Y., Li X., Mou W., Wang L., Liu Y., Reisfeld R.A., Xiang R., Lv D. (2012). SOX2 gene regulates the transcriptional network of oncogenes and affects tumorigenesis of human lung cancer cells. PLoS ONE.

[111] Laga A.C., Zhan Q., Weishaupt C., Ma J., Frank M.H., Murphy G.F. (2011). SOX2 and nestin expression in human melanoma: An immunohistochemical and experimental study. Exp. Dermatol..

[112] Büscher K., Trefzer U., Hofmann M., Sterry W., Kurth R., Denner J. (2005). Expression of human endogenous retrovirus k in melanomas and melanoma cell lines. Cancer Res..

[113] Kurth R., Bannert N. (2009). Beneficial and detrimental effects of human endogenous retroviruses. Int. J. Cancer.

[114] Conrad B., Weissmahr R.N., Boni J., Arcari R., Schupbach J., Mach B. (1997). A human endogenous retroviral superantigen as candidate autoimmune gene in type I diabetes. Cell.

[115] Young J.M., Whiddon J.L., Yao Z., Kasinathan B., Snider L., Geng L.N., Balog J., Tawil R., van der Maarel S.M., Tapscott S.J. (2013). DUX4 binding to retroelements creates promoters that are active in FSHD muscle and testis. PLOS Genet..

[116] Siebenthall K.T., Miller C.P., Vierstra J.D., Mathieu J., Tretiakova M., Reynolds A., Sandstrom R., Rynes E., Haugen E., Johnson A. (2019). Integrated epigenomic profiling reveals endogenous retrovirus reactivation in renal cell carcinoma. EBioMedicine.

[117] Manghera M., Ferguson-Parry J., Douville R.N. (2016). TDP-43 regulates endogenous retrovirus-K viral protein accumulation. Neurobiol. Dis..

[118] Nguyen T.D., Davis J., Eugenio R.A., Liu Y. (2019). Female Sex Hormones Activate Human Endogenous Retrovirus Type K Through the OCT4 Transcription Factor in T47D Breast Cancer Cells. AIDS Res. Hum. Retroviruses.

[119] Li W., Lee M.-H., Henderson L., Tyagi R., Bachani M., Steiner J., Campanac E., Hoffman D.A., von Geldern G., Johnson K. (2015). Human endogenous retrovirus-K contributes to motor neuron disease. Sci. Transl. Med..

[120] Knössl M., Löwer R., Löwer J. (1999). Expression of the human endogenous retrovirus HTDV/HERV-K is enhanced by cellular transcription factor YY1. J. Virol..

[121] Schlesinger S., Lee A.H., Wang G.Z., Green L., Goff S.P. (2013). Proviral silencing in embryonic cells is regulated by Yin Yang 1. Cell Rep..

[122] Gonzalez-Hernandez M.J., Swanson M.D., Contreras-Galindo R., Cookinham S., King S.R., Noel R., Kaplan M.H., Markovitz D.M. (2012). Expression of human endogenous retrovirus type K (HML-2) is activated by the Tat protein of HIV-1. J. Virol..

[123] Katoh I., Mírová A., Kurata S.-I., Murakami Y., Horikawa K., Nakakuki N., Sakai T., Hashimoto K., Maruyama A., Yonaga T. (2011). Activation of the long terminal repeat of human endogenous retrovirus K by melanoma-specific transcription factor MITF-M. Neoplasia.

[124] Russ E., Mikhalkevich N., Iordanskiy S. (2023). Expression of Human Endogenous Retrovirus Group K (HERV-K) HML-2 Correlates with Immune Activation of Macrophages and Type I Interferon Response. Microbiol. Spectr..

[125] Toufaily C., Landry S., Leib-Mosch C., Rassart E., Barbeau B. (2011). Activation of LTRs from different human endogenous retrovirus (HERV) families by the HTLV-1 tax protein and T-cell activators. Viruses.

[126] Stauffer Y., Marguerat S., Meylan F., Ucla C., Sutkowski N., Huber B., Pelet T., Conrad B. (2001). Interferon-alpha-induced endogenous superantigen. a model linking environment and autoimmunity. Immunity.

[127] Yu H., Liu T., Zhao Z., Chen Y., Zeng J., Liu S., Zhu F. (2014). Mutations in 3′-long terminal repeat of HERV-W family in chromosome 7 upregulate syncytin-1 expression in urothelial cell carcinoma of the bladder through interacting with c-Myb. Oncogene.

[128] Lee W.J., Kwun H.J., Kim H.S., Jang K.L. (2003). Activation of the human endogenous retrovirus W long terminal repeat by herpes simplex virus type 1 immediate early protein 1. Mol. Cells.

[129] Schön U., Seifarth W., Baust C., Hohenadl C., Erfle V., Leib-Mösch C. (2001). Cell type-specific expression and promoter activity of human endogenous retroviral long terminal repeats. Virology.

[130] Yu C., Shen K., Lin M., Chen P., Lin C., Chang G.-D., Chen H. (2002). GCMa Regulates the Syncytin-mediated Trophoblastic Fusion. J. Biol. Chem..

[131] Cherkasova E., Malinzak E., Rao S., Takahashi Y., Senchenko V.N., Kudryavtseva A.V., Nickerson M.L., Merino M., A Hong J., Schrump D.S. (2011). Inactivation of the von Hippel–Lindau tumor suppressor leads to selective expression of a human endogenous retrovirus in kidney cancer. Oncogene.

[132] Wang X., Zhao C., Zhang C., Mei X., Song J., Sun Y., Wu Z., Shi W. (2019). Increased HERV-E clone 4&ndash;1 expression contributes to DNA hypomethylation and IL-17 release from CD4^+^ T cells via miR-302d/MBD2 in systemic lupus erythematosus. Cell Commun. Signal..

[133] Sin H.-S., Huh J.-W., Kim D.-S., Kang D.W., Min D.S., Kim T.-H., Ha H.-S., Lee S.-Y., Kim H.-S. (2006). Transcriptional control of the HERV-H LTR element of the GSDML gene in human tissues and cancer cells. Arch. Virol..

[134] Katoh M., Katoh M. (2003). Identification and characterization of mouse Erbb2 gene in silico. Int. J. Oncol..

[135] Nelson D.T., Goodchild N.L., Mager D.L. (1996). Gain of Sp1 sites and loss of repressor sequences associated with a young, transcriptionally active subset of HERV-H endogenous long terminal repeats. Virology.

[136] de Parseval N., Alkabbani H., Heidmann T. (1999). The long terminal repeats of the HERV-H human endogenous retrovirus contain binding sites for transcriptional regulation by the Myb protein. J. Gen. Virol..

[137] Wang J., Xie G., Singh M., Ghanbarian A.T., Raskó T., Szvetnik A., Cai H., Besser D., Prigione A., Fuchs N.V. (2014). Primate-specific endogenous retrovirus-driven transcription defines naive-like stem cells. Nature.

[138] Wu F., Liufu Z., Liu Y., Guo L., Wu J., Cao S., Qin Y., Guo N., Fu Y., Liu H. (2022). Species-specific rewiring of definitive endoderm developmental gene activation via endogenous retroviruses through TET1-mediated demethylation. Cell Rep..

[139] Yu X., Zhu X., Pi W., Ling J., Ko L., Takeda Y., Tuan D. (2005). The long terminal repeat (LTR) of ERV-9 human endogenous retrovirus binds to NF-Y in the assembly of an active LTR enhancer complex NF-Y/MZF1/GATA-2. J. Biol. Chem..

[140] Hendrickson P.G., A Doráis J., Grow E.J., Whiddon J.L., Lim J.-W., Wike C.L., Weaver B.D., Pflueger C., Emery B.R., Wilcox A.L. (2017). Conserved roles of mouse DUX and human DUX4 in activating cleavage-stage genes and MERVL/HERVL retrotransposons. Nat. Genet..

[141] De Iaco A., Planet E., Coluccio A., Verp S., Duc J., Trono D. (2017). DUX-family transcription factors regulate zygotic genome activation in placental mammals. Nat. Genet..

[142] Mitsuhashi S., Nakagawa S., Sasaki-Honda M., Sakurai H., Frith M.C., Mitsuhashi H. (2021). Nanopore direct RNA sequencing detects DUX4-activated repeats and isoforms in human muscle cells. Hum. Mol. Genet..

[143] Chen D., Chen W., Xu Y., Zhu M., Xiao Y., Shen Y., Zhu S., Cao C., Xu X. (2019). Upregulated immune checkpoint HHLA2 in clear cell renal cell carcinoma: A novel prognostic biomarker and potential therapeutic target. J. Med. Genet..

[144] Cherkasova E., Scrivani C., Doh S., Weisman Q., Takahashi Y., Harashima N., Yokoyama H., Srinivasan R., Linehan W.M., Lerman M.I. (2016). Detection of an Immunogenic HERV-E Envelope with Selective Expression in Clear Cell Kidney Cancer. Cancer Res..

[145] Zapatka M., Borozan I., Brewer D.S., Iskar M., Grundhoff A., Alawi M., Desai N., Sültmann H., Moch H., Cooper C.S. (2020). The landscape of viral associations in human cancers. Nat. Genet..

[146] Cao W., Kang R., Xiang Y., Hong J. (2020). Human Endogenous Retroviruses in Clear Cell Renal Cell Carcinoma: Biological Functions and Clinical Values. OncoTargets Ther..

[147] Singh M., Cai H., Bunse M., Feschotte C., Izsvák Z. (2020). Human Endogenous Retrovirus K Rec Forms a Regulatory Loop with MITF that Opposes the Progression of Melanoma to an Invasive Stage. Viruses.

[148] Kitsou K., Lagiou P., Magiorkinis G. (2023). Human endogenous retroviruses in cancer: Oncogenesis mechanisms and clinical implications. J. Med. Virol..

[149] Messeguer X., Escudero R., Farré D., Núñez O., Martínez J., Albà M. (2002). PROMO: Detection of known transcription regulatory elements using species-tailored searches. Bioinformatics.

[150] Boese A., Sommer P., Holzer D., Maier R., Nehrbass U. (2009). Integrase interactor 1 (Ini1/hSNF5) is a repressor of basal human immunodeficiency virus type 1 promoter activity. J. Gen. Virol..

[151] Pisaneschi G., Ceccotti S., Falchetti M., Fiumicino S., Carnevali F., Beccari E. (1994). Characterization of FIII/YY1, a xenopus laevis conserved zinc-finger protein binding to the first exon of L1 and L14 ribosomal protein genes. Biochem. Biophys. Res. Commun..

[152] Daraiseh S.I., Kassardjian A., Alexander K.E., Rizkallah R., Hurt M.M. (2018). c-Abl phosphorylation of Yin Yang 1′s conserved tyrosine 254 in the spacer region modulates its transcriptional activity. Biochim. Biophys. Acta (BBA) Mol. Cell Res..

[153] Austen M., Luscher B., Luscher-Firzlaff J.M. (1997). Characterization of the transcriptional regulator YY1. The bipartite transactivation domain is independent of interaction with the TATA box-binding protein, transcription factor IIB, TAFII55, or cAMP-responsive element-binding protein (CPB)-binding protein. J. Biol. Chem..

[154] Shi Y., Seto E., Chang L.-S., Shenk T. (1991). Transcriptional repression by YY1, a human GLI-Krüippel-related protein, and relief of repression by adenovirus E1A protein. Cell.

[155] Shrivastava A., Calame K. (1994). An analysis of genes regulated by the multi-functional transcriptional regulator Yin Yang-1. Nucleic Acids Res..

[156] Coull J.J., Romerio F., Sun J.-M., Volker J.L., Galvin K.M., Davie J.R., Shi Y., Hansen U., Margolis D.M. (2000). The human factors YY1 and LSF repress the human immunodeficiency virus type 1 long terminal repeat via recruitment of histone deacetylase 1. J. Virol..

[157] Flanagan J.R., Becker K.G., Ennist D.L., Gleason S.L., Driggers P.H., Levi B.Z., Appella E., Ozato K. (1992). Cloning of a negative transcription factor that binds to the upstream conserved region of Moloney murine leukemia virus. Mol. Cell Biol..

[158] Satyamoorthy K., Park K., Atchison M.L., Howe C.C. (1993). The intracisternal a-particle upstream element interacts with transcription factor YY1 to activate transcription: Pleiotropic effects of YY1 on distinct DNA promoter elements. Mol. Cell. Biol..

[159] Hyde-DeRuyscher R.P., Jennings E., Shenk T. (1995). DNA binding sites for the transcriptional activator/repressor YY1. Nucleic Acids Res..

[160] Douville R.N., Nath A. (2017). Human Endogenous Retrovirus-K and TDP-43 Expression Bridges ALS and HIV Neuropathology. Front. Microbiol..

[161] Arru G., Mameli G., Deiana G.A., Rassu A.L., Piredda R., Sechi E., Caggiu E., Bo M., Nako E., Urso D. (2018). Humoral immunity response to human endogenous retroviruses K/W differentiates between amyotrophic lateral sclerosis and other neurological diseases. Eur. J. Neurol..

[162] Ding X., Xiang Z., Qin C., Chen Y., Tian H., Meng L., Xia D., Liu H., Song J., Fu J. (2021). Spreading of TDP-43 pathology via pyramidal tract induces ALS-like phenotypes in TDP-43 transgenic mice. Acta Neuropathol. Commun..

[163] Schulte A.M., Malerczyk C., Cabal-Manzano R., Gajarsa J.J., List H.-J., Riegel A.T., Wellstein A. (2000). Influence of the human endogenous retrovirus-like element HERV-E.PTN on the expression of growth factor pleiotrophin: A critical role of a retroviral Sp1-binding site. Oncogene.

[164] Aapola U., Mäenpää K., Kaipia A., Peterson P. (2004). Epigenetic modifications affect Dnmt3L expression. Biochem. J..

[165] Jaworski E., Narayanan A., Van Duyne R., Shabbeer-Meyering S., Iordanskiy S., Saifuddin M., Das R., Afonso P.V., Sampey G.C., Chung M. (2014). Human T-lymphotropic virus type 1-infected cells secrete exosomes that contain tax protein. J. Biol. Chem..

[166] Demarchi F., d’Adda di Fagagna F., Falaschi A., Giacca M. (1996). Activation of transcription factor NF-kappaB by the Tat protein of human immunodeficiency virus type 1. J. Virol..

[167] Liu J., Perkins N.D., Schmid R.M., Nabel G.J. (1992). Specific NF-kappa B subunits act in concert with Tat to stimulate human immunodeficiency virus type 1 transcription. J. Virol..

[168] Cossu D., Tomizawa Y., Sechi L.A., Hattori N. (2023). Epstein–Barr Virus and Human Endogenous Retrovirus in Japanese Patients with Autoimmune Demyelinating Disorders. Int. J. Mol. Sci..

[169] Kwun H.J., Han H.J., Lee W.J., Kim H.S., Jang K.L. (2002). Transactivation of the human endogenous retrovirus K long terminal repeat by herpes simplex virus type 1 immediate early protein 0. Virus Res..

[170] Nellåker C., Yao Y., Jones-Brando L., Mallet F., Yolken R.H., Karlsson H. (2006). Transactivation of elements in the human endogenous retrovirus W family by viral infection. Retrovirology.

[171] Hasuike S., Miura K., Miyoshi O., Miyamoto T., Niikawa N., Jinno Y., Ishikawa M. (1999). Isolation and localization of an IDDMK1,2-22-related human endogenous retroviral gene, and identification of a CA repeat marker at its locus. J. Hum. Genet..

[172] Burn A., Roy F., Freeman M., Coffin J.M. (2022). Widespread expression of the ancient HERV-K (HML-2) provirus group in normal human tissues. PLOS Biol..

[173] Coffin J.M., Hughes S.H., Varmus H.E., Coffin J.M., Hughes S.H., Varmus H.E. (1997). Retroviruses.

[174] Takahashi Y., Harashima N., Kajigaya S., Yokoyama H., Cherkasova E., McCoy J.P., Hanada K., Mena O., Kurlander R., Tawab A. (2008). Regression of human kidney cancer following allogeneic stem cell transplantation is associated with recognition of an HERV-E antigen by T cells. J. Clin. Investig..

[175] Greenig M. (2019). HERVs, immunity, and autoimmunity: Understanding the connection. PeerJ.

[176] Yin Y., Choi S.-C., Xu Z., Perry D.J., Seay H., Croker B.P., Sobel E.S., Brusko T.M., Morel L. (2015). Normalization of CD4+ T cell metabolism reverses lupus. Sci. Transl. Med..

[177] Sukapan P., Promnarate P., Avihingsanon Y., Mutirangura A., Hirankarn N. (2014). Types of DNA methylation status of the interspersed repetitive sequences for LINE-1, Alu, HERV-E and HERV-K in the neutrophils from systemic lupus erythematosus patients and healthy controls. J. Hum. Genet..

[178] Nakkuntod J., Sukkapan P., Avihingsanon Y., Mutirangura A., Hirankarn N. (2013). DNA methylation of human endogenous retrovirus in systemic lupus erythematosus. J. Hum. Genet..

[179] Wu Z., Mei X., Zhao D., Sun Y., Song J., Pan W., Shi W. (2015). DNA methylation modulates HERV-E expression in CD4+ T cells from systemic lupus erythematosus patients. J. Dermatol. Sci..

[180] Mendel D.B., Crabtree G.R. (1991). HNF-1, a member of a novel class of dimerizing homeodomain proteins. J. Biol. Chem..

[181] Anson-Cartwright L., Dawson K., Holmyard D., Fisher S.J., Lazzarini R.A., Cross J.C. (2000). The glial cells missing-1 protein is essential for branching morphogenesis in the chorioallantoic placenta. Nat. Genet..

[182] Schreiber J., Riethmacher-Sonnenberg E., Riethmacher D., Tuerk E.E., Enderich J., Bösl M.R., Wegner M. (2000). Placental failure in mice lacking the mammalian homolog of glial cells missing, GCMa. Mol. Cell. Biol..

[183] Mi S., Lee X., Li X.-P., Veldman G.M., Finnerty H., Racie L., LaVallie E., Tang X.-Y., Edouard P., Howes S. (2000). Syncytin is a captive retroviral envelope protein involved in human placental morphogenesis. Nature.

[184] Lu J., Zhang S., Nakano H., Simmons D.G., Wang S., Kong S., Wang Q., Shen L., Tu Z., Wang W. (2013). A positive feedback loop involving GCM1 and FZD5 directs chorionic branching morphogenesis in the placenta. PLOS Biol..

[185] Ohnuki M., Tanabe K., Sutou K., Teramoto I., Sawamura Y., Narita M., Nakamura M., Tokunaga Y., Nakamura M., Watanabe A. (2014). Dynamic regulation of human endogenous retroviruses mediates factor-induced reprogramming and differentiation potential. Proc. Natl. Acad. Sci. USA.

[186] Takahashi K., Nakamura M., Okubo C., Kliesmete Z., Ohnuki M., Narita M., Watanabe A., Ueda M., Takashima Y., Hellmann I. (2021). The pluripotent stem cell-specific transcript ESRG is dispensable for human pluripotency. PLOS Genet..

[187] Aronson B.E., Stapleton K.A., Krasinski S.D. (2014). Role of GATA factors in development, differentiation, and homeostasis of the small intestinal epithelium. Am. J. Physiol. Gastrointest. Liver Physiol..

[188] Ang S.L., Wierda A., Wong D., Stevens K.A., Cascio S., Rossant J., Zaret K.S. (1993). The formation and maintenance of the definitive endoderm lineage in the mouse: Involvement of HNF3/forkhead proteins. Development.

[189] Bénit L., Dessen P., Heidmann T. (2001). Identification, phylogeny, and evolution of retroviral elements based on their envelope genes. J. Virol..

[190] Pi W., Yang Z., Wang J., Ruan L., Yu X., Ling J., Krantz S., Isales C., Conway S.J., Lin S. (2004). The LTR enhancer of ERV-9 human endogenous retrovirus is active in oocytes and progenitor cells in transgenic zebrafish and humans. Proc. Natl. Acad. Sci. USA.

[191] Macfarlan T.S., Gifford W.D., Driscoll S., Lettieri K., Rowe H.M., Bonanomi D., Firth A., Singer O., Trono D., Pfaff S.L. (2012). Embryonic stem cell potency fluctuates with endogenous retrovirus activity. Nature.

[192] Sakashita A., Kitano T., Ishizu H., Guo Y., Masuda H., Ariura M., Murano K., Siomi H. (2023). Transcription of MERVL retrotransposons is required for preimplantation embryo development. Nat. Genet..

[193] Rowe W.P., Humphrey J.B., Lilly F. (1973). A major genetic locus affecting resistance to infection with murine leukemia viruses. 3. Assignment of the Fv-1 locus to linkage group 8 of the mouse. J. Exp. Med..

[194] Jolicoeur P. (1979). The Fv-1 gene of the mouse and its control of murine leukemia virus replication. Curr. Top. Microbiol. Immunol..

[195] Best S., Le Tissier P., Towers G., Stoye J.P. (1996). Positional cloning of the mouse retrovirus restriction gene Fv1. Nature.

[196] A Martens J.H., O’Sullivan R.J., Braunschweig U., Opravil S., Radolf M., Steinlein P., Jenuwein T. (2005). The profile of repeat-associated histone lysine methylation states in the mouse epigenome. EMBO J..

[197] Mikkelsen T.S., Ku M., Jaffe D.B., Issac B., Lieberman E., Giannoukos G., Alvarez P., Brockman W., Kim T.K., Koche R.P. (2007). Genome-wide maps of chromatin state in pluripotent and lineage-committed cells. Nature.

[198] Walsh C.P., Chaillet J.R., Bestor T.H. (1998). Transcription of IAP endogenous retroviruses is constrained by cytosine methylation. Nat. Genet..

[199] He Q., Kim H., Huang R., Lu W., Tang M., Shi F., Yang D., Zhang X., Huang J., Liu D. (2015). The Daxx/Atrx Complex Protects Tandem Repetitive Elements during DNA Hypomethylation by Promoting H3K9 Trimethylation. Cell Stem Cell.

[200] Leung D., Du T., Wagner U., Xie W., Lee A.Y., Goyal P., Li Y., Szulwach K.E., Jin P., Lorincz M.C. (2014). Regulation of DNA methylation turnover at LTR retrotransposons and imprinted loci by the histone methyltransferase Setdb1. Proc. Natl. Acad. Sci. USA.

[201] Imbeault M., Helleboid P.-Y., Trono D. (2017). KRAB zinc-finger proteins contribute to the evolution of gene regulatory networks. Nature.

[202] Karimi M.M., Goyal P., Maksakova I.A., Bilenky M., Leung D., Tang J.X., Shinkai Y., Mager D.L., Jones S., Hirst M. (2011). DNA methylation and SETDB1/H3K9me3 regulate predominantly distinct sets of genes, retroelements, and chimeric transcripts in mESCs. Cell Stem Cell.

[203] Gautam P., Yu T., Loh Y.-H. (2017). Regulation of ERVs in pluripotent stem cells and reprogramming. Curr. Opin. Genet. Dev..

[204] Yang P., Wang Y., Macfarlan T.S. (2017). The Role of KRAB-ZFPs in Transposable Element Repression and Mammalian Evolution. Trends Genet..

[205] Schultz D.C., Ayyanathan K., Negorev D., Maul G.G., Rauscher F.J. (2002). SETDB1: A novel KAP-1-associated histone H3, lysine 9-specific methyltransferase that contributes to HP1-mediated silencing of euchromatic genes by KRAB zinc-finger proteins. Genes Dev..

[206] Sripathy S.P., Stevens J., Schultz D.C. (2006). The KAP1 corepressor functions to coordinate the assembly of de novo HP1-demarcated microenvironments of heterochromatin required for KRAB zinc finger protein-mediated transcriptional repression. Mol. Cell. Biol..

[207] Wolf D., Goff S.P. (2007). TRIM28 mediates primer binding site-targeted silencing of murine leukemia virus in embryonic cells. Cell.

[208] Wolf D., Goff S.P. (2009). Embryonic stem cells use ZFP809 to silence retroviral DNAs. Nature.

[209] Wolf G., Yang P., Füchtbauer A.C., Füchtbauer E.-M., Silva A.M., Park C., Wu W., Nielsen A.L., Pedersen F.S., Macfarlan T.S. (2015). The KRAB zinc finger protein ZFP809 is required to initiate epigenetic silencing of endogenous retroviruses. Genes Dev..

[210] Seah M.K.Y., Wang Y., Goy P.-A.V., Loh H.M., Peh W.J., Loh D.H.P., Han B.Y., Wong E., Leong E.L., Wolf G. (2019). The KRAB-zinc finger protein ZFP708 mediates epigenetic repression at RMER19B retrotransposons. Development.

[211] Wiznerowicz M., Jakobsson J., Szulc J., Liao S., Quazzola A., Beermann F., Aebischer P., Trono D. (2007). The krüppel-associated box repressor domain can trigger de novo promoter methylation during mouse early embryogenesis. J. Biol. Chem..

[212] Wolf D., Cammas F., Losson R., Goff S.P. (2008). Primer binding site-dependent restriction of murine leukemia virus requires HP1 binding by TRIM28. J. Virol..

[213] Rowe H.M., Jakobsson J., Mesnard D., Rougemont J., Reynard S., Aktas T., Maillard P.V., Layard-Liesching H., Verp S., Marquis J. (2010). KAP1 controls endogenous retroviruses in embryonic stem cells. Nature.

[214] Brattås P.L., Jönsson M.E., Fasching L., Wahlestedt J.N., Shahsavani M., Falk R., Falk A., Jern P., Parmar M., Jakobsson J. (2017). TRIM28 Controls a Gene Regulatory Network Based on Endogenous Retroviruses in Human Neural Progenitor Cells. Cell Rep..

[215] Fasching L., Kapopoulou A., Sachdeva R., Petri R., Jönsson M.E., Männe C., Turelli P., Jern P., Cammas F., Trono D. (2015). TRIM28 represses transcription of endogenous retroviruses in neural progenitor cells. Cell Rep..

[216] Zhang W., Chen F., Chen R., Xie D., Yang J., Zhao X., Guo R., Zhang Y., Shen Y., Göke J. (2019). Zscan4c activates endogenous retrovirus MERVL and cleavage embryo genes. Nucleic Acids Res..

[217] De Iaco A., Verp S., Offner S., Grun D., Trono D. (2020). DUX is a non-essential synchronizer of zygotic genome activation. Development.

[218] Smith C.M., Grow E.J., Shadle S.C., Cairns B.R. (2023). Multiple repeat regions within mouse DUX recruit chromatin regulators to facilitate an embryonic gene expression program. bioRxiv.

[219] Whiddon J.L., Langford A.T., Wong C.-J., Zhong J.W., Tapscott S.J. (2017). Conservation and innovation in the DUX4-family gene network. Nat. Genet..

[220] Grow E.J., Weaver B.D., Smith C.M., Guo J., Stein P., Shadle S.C., Hendrickson P.G., Johnson N.E., Butterfield R.J., Menafra R. (2021). p53 convergently activates Dux/DUX4 in embryonic stem cells and in facioscapulohumeral muscular dystrophy cell models. Nat. Genet..

[221] Percharde M., Lin C.J., Yin Y., Guan J., Peixoto G.A., Bulut-Karslioglu A., Biechele S., Huang B., Shen X., Ramalho-Santos M. (2018). A LINE1-Nucleolin Partnership Regulates Early Development and ESC Identity. Cell.

[222] Zhao X., Shen J., Zhao X., Zhang M., Feng X., Zhang W., Lu X. (2022). PIM3-AMPK-HDAC4/5 axis restricts MuERVL-marked 2-cell-like state in embryonic stem cells. Stem Cell Rep..

[223] Göke J., Lu X., Chan Y.-S., Ng H.-H., Ly L.-H., Sachs F., Szczerbinska I. (2015). Dynamic transcription of distinct classes of endogenous retroviral elements marks specific populations of early human embryonic cells. Cell Stem Cell.

[224] Liu L., Leng L., Liu C., Lu C., Yuan Y., Wu L., Gong F., Zhang S., Wei X., Wang M. (2019). An integrated chromatin accessibility and transcriptome landscape of human pre-implantation embryos. Nat. Commun..

[225] Macfarlan T.S., Gifford W.D., Agarwal S., Driscoll S., Lettieri K., Wang J., Andrews S.E., Franco L., Rosenfeld M.G., Ren B. (2011). Endogenous retroviruses and neighboring genes are coordinately repressed by LSD1/KDM1A. Genes Dev..

[226] Guallar D., Perez-Palacios R., Climent M., Martinez-Abadia I., Larraga A., Fernandez-Juan M., Vallejo C., Muniesa P., Schoorlemmer J. (2012). Expression of endogenous retroviruses is negatively regulated by the pluripotency marker Rex1/Zfp42. Nucleic Acids Res..

[227] Jiang Y., Jahagirdar B.N., Reinhardt R.L., Schwartz R.E., Keene C.D., Ortiz-Gonzalez X.R., Reyes M., Lenvik T., Lund T., Blackstad M. (2002). Pluripotency of mesenchymal stem cells derived from adult marrow. Nature.

[228] Karlmark K.R., Freilinger A., Marton E., Rosner M., Lubec G., Hengstschläger M. (2005). Activation of ectopic Oct-4 and Rex-1 promoters in human amniotic fluid cells. Int. J. Mol. Med..

[229] Rogers M.B., Hosler B.A., Gudas L.J. (1991). Specific expression of a retinoic acid-regulated, zinc-finger gene, Rex-1, in preimplantation embryos, trophoblast and spermatocytes. Development.

[230] Sundaram V., Choudhary M.N.K., Pehrsson E., Xing X., Fiore C., Pandey M., Maricque B., Udawatta M., Ngo D., Chen Y. (2017). Functional cis-regulatory modules encoded by mouse-specific endogenous retrovirus. Nat. Commun..

[231] Chuong E.B., Rumi M.A.K., Soares M.J., Baker J.C. (2013). Endogenous retroviruses function as species-specific enhancer elements in the placenta. Nat. Genet..

[232] Denner J., Specke V., Thiesen U., Karlas A., Kurth R. (2003). Genetic alterations of the long terminal repeat of an ecotropic porcine endogenous retrovirus during passage in human cells. Virology.

[233] Wilson C.A., Laeeq S., Ritzhaupt A., Colon-Moran W., Yoshimura F.K. (2003). Sequence analysis of porcine endogenous retrovirus long terminal repeats and identification of transcriptional regulatory regions. J. Virol..

[234] Granadino B., Arias-De-La-Fuente C., Pérez-Sánchez C., Párraga M., López-Fernández L.A., del Mazo J., Rey-Campos J. (2000). Fhx (Foxj2) expression is activated during spermatogenesis and very early in embryonic development. Mech. Dev..

[235] van der Kuyl A.C. (2022). Analysis of Simian Endogenous Retrovirus (SERV) Full-Length Proviruses in Old World Monkey Genomes. Genes.

[236] Duan K., Si C.-Y., Zhao S.-M., Ai Z.-Y., Niu B.-H., Yin Y., Xiang L.-F., Ding H., Zheng Y. (2021). The Long Terminal Repeats of ERV6 Are Activated in Pre-Implantation Embryos of Cynomolgus Monkey. Cells.

[237] Garcia-Etxebarria K., Jugo B.M. (2014). Genomic environment and digital expression of bovine endogenous retroviruses. Gene.

[238] Halstead M.M., Ma X., Zhou C., Schultz R.M., Ross P.J. (2020). Chromatin remodeling in bovine embryos indicates species-specific regulation of genome activation. Nat. Commun..

[239] Kelly C.J., Chitko-McKown C.G., Chuong E.B. (2022). Ruminant-specific retrotransposons shape regulatory evolution of bovine immunity. Genome Res..

[240] Hierweger M.M., Koch M.C., Kauer R.V., Bagó Z., Oevermann A., Bertoni G., Seuberlich T. (2021). A novel Betaretrovirus discovered in cattle with neurological disease and encephalitis. Retrovirology.

[241] Goldstone H.M., Tokunaga S., Schlezinger J.J., Goldstone J.V., Stegeman J.J. (2012). EZR1: A novel family of highly expressed retroelements induced by TCDD and regulated by a NF-kappaB-like factor in embryos of zebrafish (Danio rerio). Zebrafish.

[242] Baur M., Esch R.K., Errede B. (1997). Cooperative binding interactions required for function of the Ty1 sterile responsive element. Mol. Cell. Biol..

[243] Madhani H.D., Fink G.R. (1997). Combinatorial control required for the specificity of yeast MAPK signaling. Science.

[244] Madison J.M., Dudley A.M., Winston F. (1998). Identification and analysis of Mot3, a zinc finger protein that binds to the retrotransposon Ty long terminal repeat (delta) in Saccharomyces cerevisiae. Mol. Cell Biol..

[245] López M.C., Baker H.V. (2000). Understanding the growth phenotype of the yeast *gcr1* mutant in terms of global genomic expression patterns. J. Bacteriol..

[246] Errede B. (1993). MCM1 binds to a transcriptional control element in Ty1. Mol. Cell Biol..

[247] Gray W.M., Fassler J.S. (1996). Isolation and analysis of the yeast *TEA1* gene, which encodes a zinc cluster Ty enhancer-binding protein. Mol. Cell. Biol..

[248] Bag I., Chen S., Rosin L.F., Chen Y., Liu C.-Y., Yu G.-Y., Lei E.P. (2021). M1BP cooperates with CP190 to activate transcription at TAD borders and promote chromatin insulator activity. Nat. Commun..

[249] Denner J., Tönjes R.R. (2012). Infection barriers to successful xenotransplantation focusing on porcine endogenous retroviruses. Clin. Microbiol. Rev..

[250] Ericsson T.A., Takeuchi Y., Templin C., Quinn G., Farhadian S.F., Wood J.C., Oldmixon B.A., Suling K.M., Ishii J.K., Kitagawa Y. (2003). Identification of receptors for pig endogenous retrovirus. Proc. Natl. Acad. Sci. USA.

[251] Patience C., Takeuchi Y., Weiss R.A. (1997). Infection of human cells by an endogenous retrovirus of pigs. Nat. Med..

[252] Halecker S., Krabben L., Kristiansen Y., Kruger L., Moller L., Becher D., Laue M., Kaufer B., Reimer C., Denner J. (2022). Rare isolation of human-tropic recombinant porcine endogenous retroviruses PERV-A/C from Gottingen minipigs. Virol. J..

[253] Specke V., Rubant S., Denner J. (2001). Productive infection of human primary cells and cell lines with porcine endogenous retroviruses. Virology.

[254] Scheef G., Fischer N., Krach U., Tönjes R.R. (2001). The number of a U3 repeat box acting as an enhancer in long terminal repeats of polytropic replication-competent porcine endogenous retroviruses dynamically fluctuates during serial virus passages in human cells. J. Virol..

[255] Denner J., Specke V., Schwendemann J., Tacke S.J. (2001). Porcine endogenous retroviruses (PERVs): Adaptation to human cells and attempts to infect small animals and non-human primates. Ann. Transplant..

[256] Jung Y.D., Lee J.R., Kim Y.J., Ha H.S., Oh K.B., Im G.S., Choi B.H., Kim H.S. (2013). Promoter activity analysis and methylation characterization of LTR elements of PERVs in NIH miniature pig. Genes Genet. Syst..

[257] Guo Y., Costa R., Ramsey H., Starnes T., Vance G., Robertson K., Kelley M., Reinbold R., Scholer H., Hromas R. (2002). The embryonic stem cell transcription factors Oct-4 and FoxD3 interact to regulate endodermal-specific promoter expression. Proc. Natl. Acad. Sci. USA.

[258] Hollister J.D., Gaut B.S. (2009). Epigenetic silencing of transposable elements: A trade-off between reduced transposition and deleterious effects on neighboring gene expression. Genome Res..

[259] Almeida L.M., Silva I.T., Silva W.A., Castro J.P., Riggs P.K., Carareto C.M., Amaral M.E.J. (2007). The contribution of transposable elements to Bos taurus gene structure. Gene.

[260] Manghera M., Magnusson A., Douville R.N. (2017). The sense behind retroviral anti-sense transcription. Virol. J..

[261] Berry B.T., Ghosh A.K., Kumar D.V., A Spodick D., Roy-Burman P. (1988). Structure and function of endogenous feline leukemia virus long terminal repeats and adjoining regions. J. Virol..

[262] Tandon R., Cattori V., Willi B., Meli M.L., Gomes-Keller M.A., Lutz H., Hofmann-Lehmann R. (2007). Copy number polymorphism of endogenous feline leukemia virus-like sequences. Mol. Cell Probes.

[263] Acevedo-Jiménez G.E., Sarmiento-Silva R.E., Alonso-Morales R.A., Córdova-Ponce R., Ramírez-Álvarez H. (2022). Detection and genetic characterization of feline retroviruses in domestic cats with different clinical signs and hematological alterations. Arch. Virol..

[264] Nesina S., Helfer-Hungerbuehler A.K., Riond B., Boretti F.S., Willi B., Meli M.L., Grest P., Hofmann-Lehmann R. (2015). Retroviral DNA—The silent winner: Blood transfusion containing latent feline leukemia provirus causes infection and disease in naïve recipient cats. Retrovirology.

[265] Tandon R., Cattori V., Pepin A.C., Riond B., Meli M.L., McDonald M., Doherr M.G., Lutz H., Hofmann-Lehmann R. (2008). Association between endogenous feline leukemia virus loads and exogenous feline leukemia virus infection in domestic cats. Virus Res..

[266] Anai Y., Ochi H., Watanabe S., Nakagawa S., Kawamura M., Gojobori T., Nishigaki K. (2012). Infectious endogenous retroviruses in cats and emergence of recombinant viruses. J. Virol..

[267] Houwing S., Kamminga L.M., Berezikov E., Cronembold D., Girard A., van den Elst H., Filippov D.V., Blaser H., Raz E., Moens C.B. (2007). A role for Piwi and piRNAs in germ cell maintenance and transposon silencing in Zebrafish. Cell.

[268] Goffeau A., Barrell B.G., Bussey H., Davis R.W., Dujon B., Feldmann H., Galibert F., Hoheisel J.D., Jacq C., Johnston M. (1996). Life with 6000 genes. Science.

[269] Curcio M.J., Garfinkel D.J. (1994). Heterogeneous functional Ty1 elements are abundant in the Saccharomyces cerevisiae genome. Genetics.

[270] Boeke J.D., Garfinkel D.J., Styles C.A., Fink G.R. (1985). Ty elements transpose through an RNA intermediate. Cell.

[271] Ribet D., Dewannieux M., Heidmann T. (2004). An active murine transposon family pair: Retrotransposition of “master” MusD copies and ETn *trans*-mobilization. Genome Res..

[272] Mager D.L., Freeman J.D. (2000). Novel mouse type D endogenous proviruses and ETn elements share long terminal repeat and internal sequences. J. Virol..

[273] Slotkin R.K., Martienssen R. (2007). Transposable elements and the epigenetic regulation of the genome. Nat. Rev. Genet..

[274] Turkel S., Liao X.B., Farabaugh P.J. (1997). GCR1-dependent transcriptional activation of yeast retrotransposon Ty2-917. Yeast.

[275] Morillon A., Springer M., Lesage P. (2000). Activation of the Kss1 invasive-filamentous growth pathway induces Ty1 transcription and retrotransposition in *Saccharomyces cerevisiae*. Mol. Cell. Biol..

[276] Ciriacy M., Freidel K., Löhning C. (1991). Characterization of trans-acting mutations affecting Ty and Ty-mediated transcription in Saccharomyces cerevisiae. Curr. Genet..

[277] Company M., Errede B. (1987). Cell-type-dependent gene activation by yeast transposon Ty1 involves multiple regulatory determinants. Mol. Cell. Biol..

[278] Grant P.A., Duggan L., Côté J., Roberts S.M., Brownell J.E., Candau R., Ohba R., Owen-Hughes T., Allis C.D., Winston F. (1997). Yeast Gcn5 functions in two multisubunit complexes to acetylate nucleosomal histones: Characterization of an Ada complex and the SAGA (Spt/Ada) complex. Genes Dev..

[279] Kent N.A., Karabetsou N., Politis P.K., Mellor J. (2001). In vivo chromatin remodeling by yeast ISWI homologs Isw1p and Isw2p. Genes Dev..

[280] Laloux I., Dubois E., Dewerchin M., Jacobs E. (1990). TEC1, a gene involved in the activation of Ty1 and Ty1-mediated gene expression in Saccharomyces cerevisiae: Cloning and molecular analysis. Mol. Cell. Biol..

[281] Morillon A., Bénard L., Springer M., Lesage P. (2002). Differential effects of chromatin and Gcn4 on the 50-fold range of expression among individual yeast Ty1 retrotransposons. Mol. Cell. Biol..

[282] Pollard K.J., Peterson C.L. (1997). Role for *ADA/GCN5* products in antagonizing chromatin-mediated transcriptional repression. Mol. Cell. Biol..

[283] Servant G., Pinson B., Tchalikian-Cosson A., Coulpier F., Lemoine S., Pennetier C., Bridier-Nahmias A., Todeschini A.L., Fayol H., Daignan-Fornier B. (2012). Tye7 regulates yeast Ty1 retrotransposon sense and antisense transcription in response to adenylic nucleotides stress. Nucleic Acids Res..

[284] Roeder G.S., Farabaugh P.J., Chaleff D.T., Fink G.R. (1980). The origins of gene instability in yeast. Science.

[285] Curcio M.J., Lutz S., Lesage P. (2015). The Ty1 LTR-Retrotransposon of Budding Yeast, *Saccharomyces cerevisiae*. Microbiol. Spectr..

[286] Lydall D., Ammerer G., Nasmyth K. (1991). A new role for MCM1 in yeast: Cell cycle regulation of SW15 transcription. Minerva Anestesiol..

[287] Gray W.M., Fassler J.S. (1993). Role of Saccharomyces cerevisiae Rap1 protein in Ty1 and Ty1-mediated transcription. Gene Expr..

[288] Tubio J.M., Tojo M., Bassaganyas L., Escaramis G., Sharakhov I.V., Sharakhova M.V., Tornador C., Unger M.F., Naveira H., Costas J. (2011). Evolutionary dynamics of the Ty3/gypsy LTR retrotransposons in the genome of Anopheles gambiae. PLoS ONE.

[289] Mager D.L., Stoye J.P. (2015). Mammalian Endogenous Retroviruses. Microbiol. Spectr..

